# Overrepresentation Bias Leads to Performance Overestimation in Blood–Brain Barrier Permeability Prediction Models: Characterization and Mitigation

**DOI:** 10.1021/acs.jcim.5c02891

**Published:** 2026-06-02

**Authors:** Pablo Ferri, Juan M. García-Gómez

**Affiliations:** Biomedical Data Science Laboratory (BDSLab), Instituto de Aplicaciones de las Tecnologías de la Información y de las Comunicaciones Avanzadas (ITACA), Universitat Politècnica de València (UPV), Camí de Vera s/n, València 46022, Spain

## Abstract

Recent advancements in blood–brain barrier permeability (BBBP) prediction of drug compounds have highlighted the growing role of machine learning, particularly deep learning. While considerable attention has been given to feature engineering and model design, their evaluation often receives insufficient attention despite its fundamental role in model credibility. In this work, we study a phenomenon we term *overrepresentation bias*, susceptible to be found in drug property databases, characterized by the presence of near-identical compounds with the same or nearly identical property values. Our findings reveal that overrepresentation bias leads to overly optimistic performance estimates in BBBP prediction models by significantly inflating test evaluation metrics—13.3% in average for the area under curve and 16.44% in average for the macro F1-score. To address this bias, we propose (i) an automatic detection algorithm and (ii) a bias-aware data handling procedure. We recommend adopting this approach to ensure more reliable model evaluations. Given that overrepresentation bias can affect performance estimation more than feature selection, model architecture, or even training data, we urge both academic and industrial communities to acknowledge its significance and take proactive measures to identify and address this bias in future studies.

## Graphical abstract

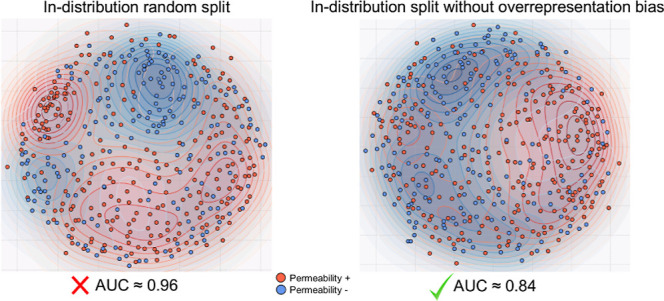

## 1 Introduction

The integration of machine learning, particularly deep learning, into drug discovery pipelines has revolutionized the prediction of molecular properties and the design of novel compounds.^1^ These computational models leverage large chemical data sets to identify patterns that inform early-stage drug discovery, facilitating the elucidation of structure–activity relationships, predicting physicochemical properties, and guiding compound optimization.^2^ As a result, these technologies have the potential to reduce the time and cost of drug development while improving the success rates of candidate compounds.

Recent advances in molecular data representation and the development of sophisticated models—particularly deep learning architectures—have significantly enhanced the ability to decipher complex chemical patterns and relationships.^3–6^ These technological strides hold immense promise for predicting critical properties such as solubility, toxicity, and permeability with high accuracy.^7^ Additionally, the number of publicly available repositories for model development and validation continues to expand,^8^ further accelerating progress in the field. Together, these factors contribute to an optimistic outlook for machine learning in drug discovery.

However, despite notable successes, several critical challenges remain. While substantial efforts have been devoted to optimizing chemical data representations and developing increasingly sophisticated architectures, rigorous model evaluation has received comparatively little attention. Much of the field continues to emphasize refinements to deep learning models that often deliver only incremental gains, while overlooking the decisive influence of test data selection on the reported performance. Clear and appropriate inclusion criteria for test data sets are essential to ensure that evaluation metrics capture a model’s true predictive capabilities rather than artifacts of data set composition. Without such rigor, models that appear to achieve exceptional results may in fact perform considerably worse in real-world applications, leading to inflated performance estimates and misleading conclusions about predictive capabilities.

In this context, we identify and examine a data set bias susceptible to be found in drug property databases, which we term *overrepresentation bias*. This bias arises when data sets include multiple near-identical compounds with identical or nearly identical property values. If data preprocessing does not adequately address this issue, then it can lead to artificially inflated performance estimates during model evaluation. Such redundancy may create the illusion of superior predictive performance as models may memorize these overrepresented instances rather than learning meaningful, generalizable patterns. As a result, true model-predictive capabilities are compromised, undermining its reliability in practical applications.

In this study, we investigate the impact of overrepresentation bias on the prediction of blood–brain barrier permeability (BBBP) of drug compounds, providing a detailed analysis of its effects. The BBB is a highly specialized interface that separates the central nervous system (CNS) from systemic circulation. It plays a crucial role in regulating the brain’s internal environment by selectively permitting the entry of essential nutrients while restricting potentially harmful substances and xenobiotics.^9^ This selective permeability is essential for maintaining neural homeostasis but poses a significant challenge in the development of CNS-targeted therapeutics.^10^ Accurately predicting BBB permeability is a key objective in modern drug development, as CNS-active drugs must successfully traverse this barrier, while unintended penetration by non-CNS-targeted drugs can result in adverse neurotoxic effects.^11^ Consequently, early and accurate prediction of BBB permeability has become an essential step in screening and optimizing candidate molecules.^12^

We focus on this problem because of its high clinical relevance and the increasing number of studies reporting near-perfect performance using novel machine learning models. Through an exhaustive analysis of data set composition and the implementation of rigorous evaluation protocols, we demonstrate how the presence of near-identical compounds can significantly distort performance metrics and why performance estimates should be interpreted more conservatively. Our findings reveal that models evaluated on biased BBB permeability data sets exhibit an artificially high predictive performance. This discrepancy underscores the urgent need for unbiased evaluation strategies to ensure reliable assessments of the model predictive capabilities.

This work highlights the critical importance of addressing overrepresentation bias in the BBB permeability prediction models. Moreover, we caution that this issue may extend beyond BBB prediction, affecting other drug property prediction tasks, particularly as the increasing availability of open data sets and their inherent data structures makes these problems prone to overrepresentation bias. By bringing attention to this often-overlooked issue, we aim to promote good practices that enhance the accuracy, robustness, and trustworthiness of predictive models. Ultimately, addressing these challenges is essential for advancing machine intelligence in drug discovery and ensuring that computational innovations translate to meaningful real-world applications.

## 2 Background

### 2.1 Theoretical Background

We introduce a series of definitions essential for understanding the analyses presented in this work. For these definitions, we assume that the data comes from drug property databases—although the concepts can be extended to other domains—and that these properties can be expressed as either categorical or numerical values.

Definition 1 (Near-duplicate compounds). Two chemical compounds are considered near-duplicates if they exhibit high structural similarity but differ by minor modifications.
Definition 2 (Exact feature collision). A phenomenon in which distinct chemical compounds share the exact same numerical representation within a specific feature space.
Definition 3 (Approximate feature collision). A circumstance in which distinct chemical compounds have nearly identical numerical representations within a specific feature space.

Figure 1 exemplifies the concepts presented above.

Definition 4 (Near-duplicate instances). Near-duplicate compounds that exhibit the same property values if the property is categorical or identical or nearly identical values if the property is numerical.
Definition 5 (Overrepresentation bias). A type of data set bias arising from the excessive presence of near-duplicate instances within a data set. These instances are disproportionately represented relative to their actual prevalence or significance in the real-world context in which the machine learning model is intended to operate.

**Figure 1:**
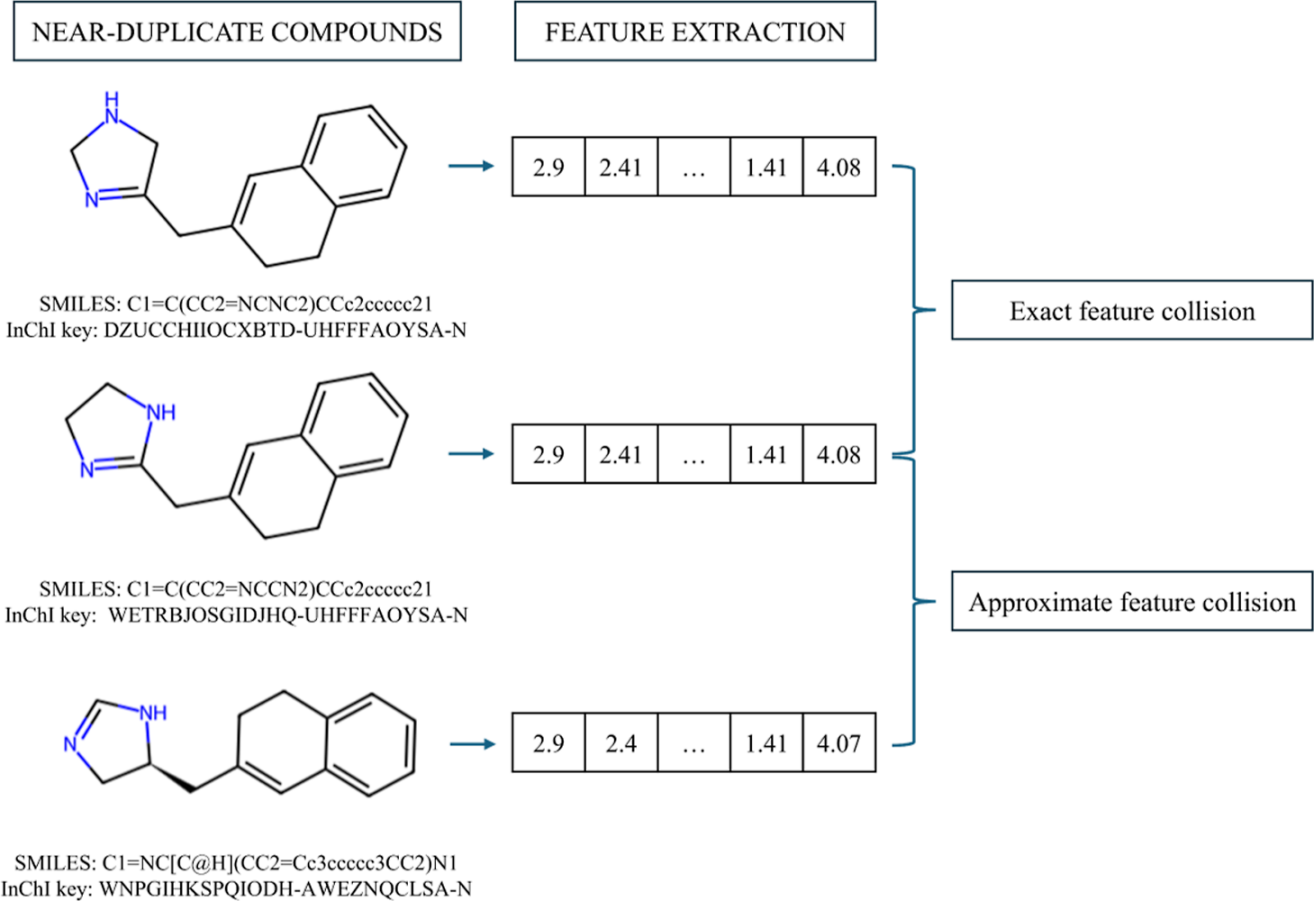
Exemplification of key concepts in this study: near-duplicate compounds, exact feature collision, approximate feature collision. Abbreviations: SMILES, simplified molecular input line entry system; InChI, international chemistry identifier.

### 2.2 BBB Permeability Prediction

In this work, we focus on predicting BBB permeability using machine learning to assess the impact of overrepresentation bias. Table 1 presents the most relevant recent studies on BBB permeability prediction through machine learning, summarizing the input features used, models considered, and reported performance metrics.

**Table 1:** Previous BBB Permeability Machine Learning Models^a^

reference	inputs	model	AUC	Acc	recall	Spec
^13^	MolDesc	gradient boosting	0.94	0.89	0.93	0.77
 ^14^	MolDesc Fingerprints	FeedFwd	0.977	0.971	0.974	0.984
 ^15^	MolDesc Fingerprints	FeedFwd	0.992	0.981	0.988	0.959
 ^16^	MolDesc Fingerprints SMILES Molecule images	Multimodal deep learning (FeedFwd,LSTM,convolutional)	0.83	0.74	0.85	0.64
 ^17^	MolDesc Fingerprints	FeedFwd	0.98	0.978	0.97	0.98
 ^18^	MolDesc	gradient boosting	n/a	0.85	0.42	0.99
 ^19^	MolDesc	linear discriminant analysis	0.92	0.851	0.88	n/a
^20^	SMILES	MegaMolBART + Gradient boosting	0.88	n/a	n/a	n/a
^21^	MolDesc	gradient boosting	0.971	0.912	n/a	n/a

a Abbreviations: AUC., area under curve; Acc., accuracy; Spec., specificity; MolDesc., molecular descriptors; FeedFwd., feed forward deep neural network; LSTM., Long short-term memory.

Table 1 shows that the primary input features for this task are molecular descriptors, molecular fingerprints, and SMILES representations.^22^ The most prominent models are based on gradient boosting and deep learning. The reported performance varies considerably across studies, with some achieving high values, while others present more moderate results, thus indicating significant variability in the reported metrics.

## 3 Materials

### 3.1 Source Repositories

We collected data from publicly recognized benchmark repositories and recent studies that openly shared their working data sets. These sources compile data from more than 50 previous studies, with each repository integrating information from multiple sources. Table 2 summarizes the data sets used. By aggregating these distinct repositories, we aimed to construct a comprehensive representation of the publicly available BBBP chemical space, ensuring that our bias analysis is robust across different data origins. All compounds were available in the SMILES format, and each included a label value indicating whether the compound was BBB-permeable or not.

**Table 2:** Multi-Source Repositories Considered in this Work^a^

repository identifier	number compounds	positive permeability frequency (%)	reference
MoleculeNet	2050	76.44	^23^
B3DB	7807	63.48	^24^
DeePred-BBB	3605	72.32	^15^
QSAR-BBB	1004	51.79	^25^
NeuTox2	12,903	62.3	^21^

a The dataset sizes for each repository, as well as the relative number of positive cases, are presented.

It is important to note that the permeability labels provided by the source repositories (Table 2) reflect the net transport outcome rather than a specific mechanism (e.g., passive diffusion, active transport). The nonpermeable class generally encompasses both compounds with poor passive membrane permeability and those that are effective substrates for efflux transporters. Consequently, the predictive models developed in this study are trained to forecast the aggregate phenotype of BBBP, capturing the combined effects of physicochemical properties and transporter interactions implicit in the training data.

We merged the five multisource repositories considered in this study. To ensure the highest level of chemical integrity and resolve ambiguities arising from noncanonical, isomeric, and tautomeric SMILES representations, we employed a rigorous curation pipeline utilizing RDKit. Specifically, we incorporated the structural standardization and detautomerization workflows inspired by the trusted protocols proposed by ref 26. The automated pipeline proceeded as follows:

1. Validation: We parsed the initial SMILES strings and applied a fragment chooser algorithm to retain only the largest organic fragment, effectively stripping disconnected salts and solvent molecules.
2. Charge Neutralization: We neutralized the remaining molecular fragments, standardizing the charge states by adding or removing hydrogens where structurally appropriate.
3. Structural normalization and detautomerization: Recognizing that tautomers are interconvertible chemical forms that can artificially inflate data set redundancy, we applied a tautomer enumerator algorithm. This step evaluates all possible tautomeric states and canonicalizes the molecule to its most stable or standard form on the basis of empirical scoring rules, unifying functionally identical compounds.
4. Standardized SMILES Generation: Following detautomerization, we exported these normalized molecules back into canonical, isomeric SMILES strings. These *Fourches-standardized* SMILES served as our definitive ground truth for the molecular identity.
5. Strict Deduplication and Conflict Resolution: We identified and removed all duplicate instances defined by matching standardized SMILES strings, ensuring that no single chemical entity (or its interconvertible tautomeric forms) was represented more than once. Finally, we eliminated cases in which identical canonical structures were associated with conflicting biological permeability labels.

This standardized curation process reduced the initial aggregated data set from 27,369 entries to 7845 unique, canonical compounds. Thus, the analyses presented in this work are performed on a strictly curated chemical data set, ensuring that the reported overrepresentation bias is entirely attributable to the similarity between distinct compounds in the feature space, rather than tautomeric redundancies or identical data duplication errors.

The source data, along with the processed data presented in later sections of this study, can be found on our GitHub repository.

### 3.2 Framework

The implementation language of our experiments was Python,^27^ using the libraries RDKit,^28^ Numpy,^29^ and Pandas^30^ for data management. To implement and train the designed models, we considered scikit-learn,^31^ PyTorch,^32^ and HuggingFace’s Transformers.^33^ Finally, we used Optuna^34^ for hyperparameter tuning.

## 4 Methods

### 4.1 Feature Extraction

We present below the input features derived for building our BBB permeability prediction models:

#### 4.1.1 Molecular Descriptors

Given the extensive use of molecular descriptors in previous BBB permeability studies (see Table 1), we included them as predictive features. We calculated a comprehensive set of descriptors using Mordred,^35^ then removed any variables with zero variance—discarding uninformative constants and reducing potential numerical instabilities. The resulting matrix comprised 709 numerical molecular descriptors.

To ensure numerical stability and consistency across varying scales, we normalized these descriptors using robust scaling. For each descriptor *j*, we computed its median (med_*j*_) and interquartile range (IQR_*j*_) and applied

1 $$x_{ij}^{*}=\frac{x_{ij}-{med}_{j}}{{IQR}_{j}}$$

where *x*_*ij*_ is the original value and *x*_ij_^*^ is the normalized value.

We performed normalization after data partitioning to prevent information leakage in the test set. Since normalization parameters are calculated from the underlying data distribution, applying this step before splitting would compromise the reliability of our results. Our data partitioning method is explained in detail in Section 4.3 (*Overrepresentation-aware Data Splitting*).

#### 4.1.2 Molecular Access System (MACCS) Fingerprints

We considered the 166 bit molecular access system (MACCS) structural-key fingerprint—a fixed-length binary vector in which each bit encodes a predefined substructure pattern.^36^ MACCS keys are particularly well suited for BBB permeability studies because they combine computational parsimony—the short, collision-free vector reduces memory requirements and speeds model training—with demonstrated utility: they have been widely adopted in BBB permeability prediction and related modeling tasks (see Table 1).

We also extracted the indices of nonzero bits from each MACCS fingerprint. These indices were used as inputs for some models, while others utilized the full-fingerprint vectors directly. Specific algorithms, implementation details, and the method by which each approach used these MACCS representations are described in Section 4.4 (*Modeling*).

#### 4.1.3 Extended-Connectivity Fingerprints

We employed extended-connectivity fingerprints (ECFPs) as input features due to their rich structural representation, which effectively captures diverse local molecular features. ECFP offers several advantages, including rotational invariance, computational efficiency, and widespread adoption in BBB permeability prediction (see Table 1) and other molecular property prediction tasks.^37^

Analogous to our treatment of MACCS fingerprints, we extracted the indices of the nonzero positions from each ECFP vector. These indices served as inputs for some models, whereas others directly used a full fingerprint vector. The specific models and their implementations are detailed in Section 4.4 (*Modeling*).

#### 4.1.4 Graph Features

We derived the graph structure for each molecule, capturing both the connectivity pattern and the relevant atomic and bond features. For atomic features, we included atomic number, hybridization, partial charge, electronegativity, covalent radius, van der Waals radius, degree, and aromaticity, as these properties have been shown to be informative in previous studies.^38^ Additionally, each graph edge was characterized by its bond type.

To ensure consistency in numerical atom feature values, we applied the appropriate normalization techniques. Specifically, we used a logarithmic transformation for electronegativity, covalent radius, and van der Waals radius. The degree was normalized by dividing it by its maximum possible value, which we set to 12, accounting for cases involving heavy metals. This approach aligns with the normalization strategy applied to molecular descriptors, ensuring that numerical atom features remain within a comparable range and thereby improving numerical stability and facilitating model convergence.

#### 4.1.5 SMILES Tokens

From the original SMILES strings, we first derived canonical SMILES^22^ to ensure a single, standardized representation for each molecule, thereby resolving the ambiguity that arises from multiple possible notations. Although canonical SMILES provide consistency, they encode chemical information in a single linear representation that may not explicitly capture important molecular substructures. To overcome this limitation, we subsequently transformed canonical SMILES into SMILES tokens. SMILES tokens are discrete chemical fragments extracted from canonical SMILES, explicitly representing atoms, functional groups, branches, and ring structures as separate tokens. This fragmentation results in a richer and more interpretable representation of the molecular structure, facilitating more effective learning and generalization by machine learning models. To accurately generate these chemically meaningful tokens, we employed a specialized chemical tokenizer, specifically, the ChemBERTa tokenizer, given its demonstrated effectiveness in previous chemoinformatics studies.^39^

### 4.2 Near-Duplicate Compound Detection

In this section, we describe the methodology for categorizing pairs of chemical compounds as near duplicates. Compounds were deemed near-duplicates if they exhibited either exact or approximate feature collisions. To detect these collisions, we took as feature representation ECFP, introduced in Section 4.1 (*Feature Extraction*).

We acknowledge that relying on 2D fingerprints such as ECFP implies a loss of information regarding 3D conformation and stereoisomerism compared to high-fidelity quantum mechanical representations. However, ECFP was selected as the metric for bias detection for two key reasons:

1. Connectivity Awareness: Contrary to simple substructure keys, ECFP explicitly encodes local topological connectivity through circular atom environments, making it a robust proxy for structural similarity in the absence of experimental 3D coordinates.
2. Model-Centric Bias Detection: The primary objective of this study is to quantify the performance inflation of the machine learning models. Since a significant proportion of the state-of-the-art BBBP models rely on 2D fingerprints or SMILES (Table 1), analyzing bias within this specific feature space is essential. If two compounds exhibit feature collision, then the model treats them as identical, regardless of their differences in a higher dimensional space. Therefore, measuring overrepresentation in the ECFP space provides a diagnosis of the redundancy that directly affects the model evaluation.

#### 4.2.1 Exact Feature Collision Detection

To detect exact feature collisions, we identified pairs of compounds with distinct molecular structures—as confirmed by different *Fourches-standardized* SMILES—that yielded identical ECFP fingerprints. We computed the Jaccard distance^40^ between each pair of ECFP vectors, **x**_*i*_ and **x**_*j*_, as

2 $$d_{J}\left(x_{i},x_{j}\right)=1-\frac{\left|x_{i}\cap x_{j}\right|}{\left|x_{i}\cup x_{j}\right|}$$

where |**x**_***i***_ **∩ x**_***j***_| represents the number of features they have in common. |**x**_***i***_ **∪ x**_***j***_| represents the total number of unique features found in either set.

Thus, any pair with *d*_J_ = 0 was classified as a near-duplicate due to exact feature collision.

#### 4.2.2 Approximate Feature Collision Detection

To detect approximate feature collisions, we again employed a distance-based criterion: any two distinct compounds whose Jaccard ECFP-based distance fell at or below a threshold τ were considered near-duplicate compounds.

Because the choice of τ critically determines which pairs qualify as approximate collisions, we developed an automatic, data-driven procedure to set it

1. Nearest-Neighbor Distance Extraction

After filtering out exact-collision near-duplicates, we compute, for each compound, the distance to its closest neighbor. We focus on these distances because they:

2 Modeling the Nearest-Neighbor Distance Distribution

(a) capture each compound’s local similarity structure and
(b) require far fewer computations than examining all pairwise distances.

We assume that the empirical distribution of these distances follows one of four patterns:

- Single beta distribution. A unimodal density for *D*, the nearest-neighbor distance:

3 D∼Beta(α,β)

where α, β are the shape parameters of the Beta distribution.

- Two-component beta mixture (left-heavy). A dominant left-hand peak representing the typical distances, and a smaller right-hand peak corresponding to divergent compounds that may include outliers:

4 $$D∼\omega Beta\left(\alpha_{1},\beta_{1}\right)+\left(1-\omega \right)Beta\left(\alpha_{2},\beta_{2}\right),,0\leq \omega \leq 1$$

where ω is the mixture weight of the primary component; α_1_, β_1_ and α_2_, β_2_ are the shape parameters of the two Beta distributions.

- Two-component beta mixture (right-heavy). A dominant right-hand peak for typical distances and a smaller left-hand peak for near-duplicates:

5 $$D∼\left(1-\omega \right)Beta\left(\alpha_{1},\beta_{1}\right)+\omega Beta\left(\alpha_{2},\beta_{2}\right),,0\leq \omega \leq 1$$

where, in this case, the smaller left-hand component (Beta­(α_1_, β_1_)) captures the cluster of small distances indicative of approximate collisions.

- Three-component beta mixture. A central peak for typical distances flanked by peaks for approximate collisions and divergent compounds:

6 $$D∼\sum_{j=1}^{3}\omega_{j}Beta\left(\alpha_{j},\beta_{j}\right),,\omega_{j}\geq 0,\sum_{j=1}^{3}\omega_{j}=1$$

each ω_*j*_ is the mixture weight of one component and α_*j*_, β_*j*_ are its shape parameters.

Regarding the estimation of the mixture weights {ω_*j*_} and the shape parameters {α_*j*_, β_*j*_}, we employed the expectation–maximization (EM) algorithm.^41^ Since standard EM can produce results that are mathematically optimal but practically useless (e.g., two component curves that are almost identical), we incorporated two penalty terms into the objective function, acting as soft optimization constraints. The objective of each penalty was as follows:

1. Reduce overlap between components. We quantify the overlap between each pair of Beta components (*j* and *k*) using the following expression, which calculates the overlap coefficient, $O_{jk}$ :

7 $$O_{jk}=\int_{0}^{1}\;\min\left(f_{j}\left(d\right),f_{k}\left(d\right)\right)dd$$

where *d* represents a specific value of the Jaccard distance, and *f*_*j*_(*d*) and *f*_*k*_(*d*) are the respective Beta-density functions for components *j* and *k*. The integral calculates the total area of the region shared by both density curves. Hence, minimizing this overlap encourages well-separated mixture components.

2. Enforce an ordering on component means. To ensure the mixture components are identifiable, we enforce a monotonically increasing order on their means. The mean of a Beta component *j*, denoted by μ_*j*_, is defined as

8 $$\mu_{j}=\frac{\alpha_{j}}{\alpha_{j}+\beta_{j}}$$

To impose the desired ordering, μ_1_ < μ_2_ <···< μ_*K*_ (whenever those components are present), we introduce the following penalty term to the objective function:

Let $\mu_{j}=\frac{\alpha_{j}}{\alpha_{j}+\beta_{j}}$ denote the mean of component *j*. We add a penalty

9 $$P_{order}=\sum_{1\leq j<k\leq K}\max\left(0,\mu_{j}-\mu_{k}\right)$$

This penalty functions as a soft constraint. If any pair of component means is in the correct order (i.e., μ_*j*_ < μ_*k*_ for *j* < *k*), the term μ_*j*_ – μ_*k*_ is negative, and the max­(0, ...) function evaluates to zero, adding no penalty. Conversely, if the means are disordered (μ_*j*_ > μ_*k*_), the term is positive, incurring a penalty that the optimization algorithm seeks to minimize. This effectively guides the model parameters toward a solution that respects the specified ordering.

As mentioned before, model parameters are fit using an iterative EM framework. In the expectation step, we determine the soft assignment of each data point to every component by calculating the responsibilities *r*_*ij*_

10 $$r_{ij}=\frac{\omega_{j}f_{j}\left(d_{i}\right)}{\sum_{k=1}^{K}\omega_{k}f_{k}\left(d_{i}\right)}$$

This value, *r*_*ij*_, represents the probability that a given distance *d*_*i*_ belongs to the component *j*.

In the subsequent maximization step, we update the model’s parameters by minimizing a penalized objective function, $L_{pen}$ . This function is designed to find parameters that not only fit the data well but also adhere to the two soft constrains presented before

11 $$L_{pen}=-\sum_{i=1}^{n}\sum_{j=1}^{K}r_{ij}\;\ln f_{j}\left(d_{i}\right)+\lambda_{ov}\sum_{1\leq j<k\leq K}O_{jk}+\lambda_{pl}\sum_{1\leq j<k\leq K}\max\left(0,\mu_{j}-\mu_{k}\right)$$

The influence of the penalty terms is controlled by their respective positive penalty weights, λ_ov_ and λ_pl_. During each M-step, we minimize $L_{pen}$ using an inner L-BFGS-B routine.^42^

After fitting each of the four candidate distributions, we select the best model according to the Bayesian information criterion (BIC)^43^

12 $$BIC=-2\;\ln\left(L_{pen}\left(\theta^{*}\right)\right)+p\;\ln n$$

where θ* denotes the optimized parameters, *p* is the number of free parameters, and *n* is the sample size.

We performed all model fitting and hyperparameter tuning on a training set from which exact feature duplicates had been removed; this overrepresentation-aware data splitting procedure is detailed in Section 4.3. The hyperparameters—such as the number of EM iterations and the penalty weights λ_ov_, λ_pl_—were optimized using a Bayesian approach with expert-defined priors within a k-fold cross-validation framework. To prevent data leakage, the test set was held out during this entire process.

Finally, if the optimal model was a two-component (right-heavy) or a three-component mixture—the two cases that identify the presence of approximate collisions—we computed the automatic threshold τ by finding d* ∈ (0, 1) such that

13 $$\omega_{1}f_{1}\left(d^{*}\right)=\omega_{2}f_{2}\left(d^{*}\right)$$

i.e., where the density of the near-duplicates component *f*_1_(*d*) equals that of the standard distances component *f*_2_(*d*). We solve this equation numerically using Brent’s method^44^ and set τ = d*.

### 4.3 Overrepresentation-Aware Data Splitting

To evaluate the impact of overrepresentation bias on our machine learning models, we developed a multistage data splitting and filtering procedure. This process creates three parallel sets of training and testing data, each with a progressively stricter definition of the compound similarity.

#### 4.3.1 Train and Test Splits

##### 4.3.1.1 Stage 1: Initial Stratified Random Split

First, we split our data set into two sets: the *random train set* and the *random test set*, with proportions of 75% and 25%, respectively. The data was shuffled before splitting to avoid introducing biases associated with its initial order. Additionally, a stratified partitioning strategy was used to ensure that class label proportions remained consistent across both splits.

##### 4.3.1.2 Stage 2: Filtering Exact Feature Collisions

For the *random training set*, we inspected and removed exact feature collisions. When collisions occurred with the same label, we retained only the first instance. When collisions involved discrepant labels, we removed the affected instances to avoid the introduction of noise. We named the resulting training set as the *exact training set*.

Then, we applied the same filtering process to the *full random test set*, inspecting the presence of feature collisions. When instances shared the same label, we kept the first occurrence. When instances had conflicting labels, then they were removed. This process resulted in the *full-exact raw test*.

After filtering for exact collisions, we compared the *full exact raw test set* with the *exact train set* and removed any instances in the test set that presented exact collisions with the training set. This process resulted in the *full exact test set*.

##### 4.3.1.3 Stage 3: Filtering Approximate Feature Collisions

The next step involved filtering both the *exact training set* and the *full exact test set* to detect and handle approximate feature collisions. If collisions shared the same true label, then we kept only the first instance. If the labels were discrepant, unlike in the exact collision case, we retained them as these permeability discrepancies could be informative instead of noise. The result of this step was the *exact and approximate (ExApr) training set* and the *raw ExApr test set*.

We then compared the *raw ExApr test set* with the *ExApr train set* to remove near-duplicates already present in the training set. If labels agreed, the instance was removed from the test set. If labels conflicted, we retained the instance.

##### 4.3.1.4 Stage 4: Harmonizing Test Sets for Fair Evaluation

Finally, we extracted the number of instances in the *ExApr Test Set* as well as the class frequencies. To ensure that all three test sets (random, exact, and ExApr) contained the same number of compounds and exhibited the same class frequencies, we selected a stratified sample from the *full random* and *exact test sets* that matched the size and frequency distribution of the *ExApr test set*.

Figure 2 summarizes the data splitting and filtering process, detailing the final data count and positive class frequency after each of the operations described above.

**Figure 2:**
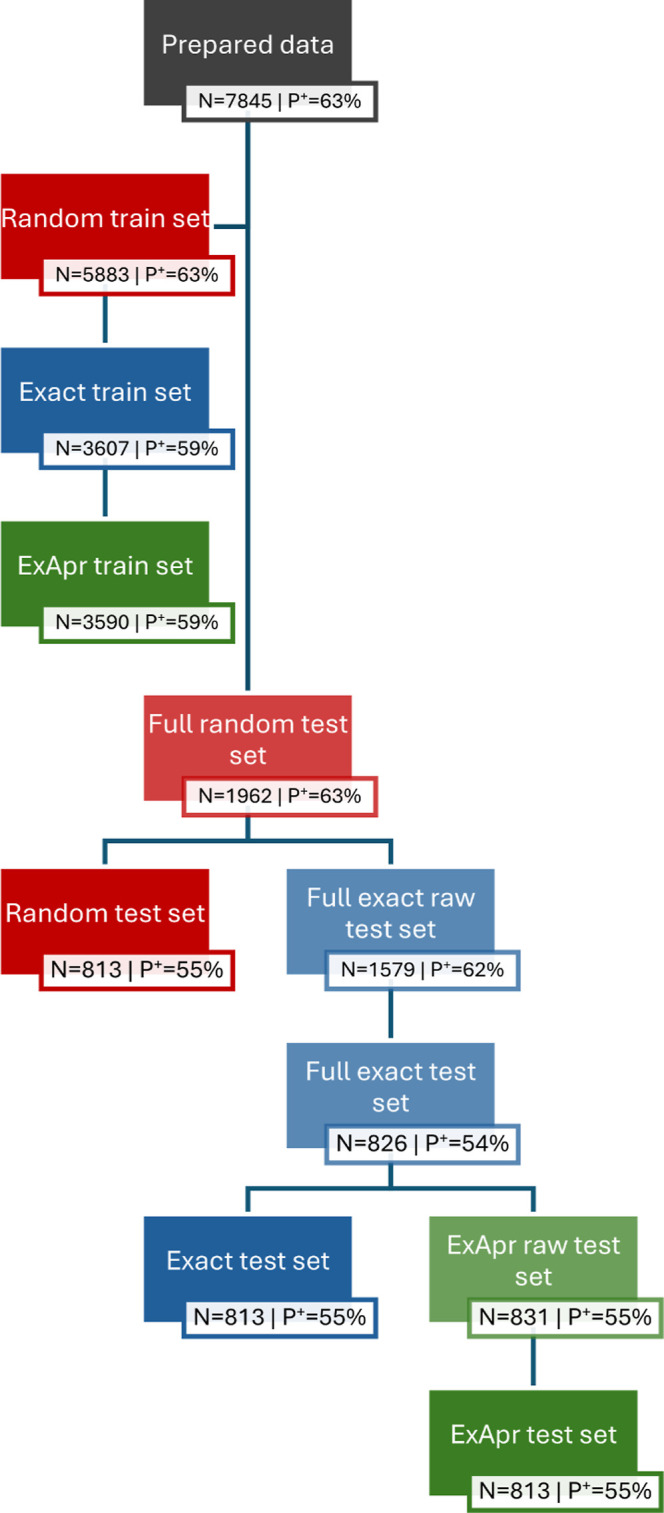
Overrepresentation-aware data splitting summary. Abbreviations: ExApr, exact and approximate; N., number of data; P^+^, positive blood-brain barrier permeability frequency.

#### 4.3.2 Cross-Validation Splits

After splitting the data into training and test sets, we further divided each training data set—random, exact, and ExApr—into four nonoverlapping K-folds. We chose four folds as a trade-off between computational cost and obtaining a reliable estimate of the prediction error. For each fold, 75% of the data was used exclusively for training, while the remaining 25% was reserved for validation. This partitioning was specifically conducted for hyperparameter optimization.

### 4.4 Modeling

To assess the impact of overrepresentation bias on BBB permeability prediction, we implemented a range of predictive models known for their strong performance in this and other property prediction tasks (see Table 1 ^45,46^ ). Each model is described below, along with its technical justification.

#### 4.4.1 Random Forest

Random forest is an ensemble learning method that constructs a multitude of decision trees and aggregates their outputs to capture complex, nonlinear relationships within the data. By randomly selecting subsets of features at each split, this model primarily reduces variance, thereby mitigating the risk of overfitting.^47^ In our study, random forests were applied to two distinct input representations: molecular descriptors and fingerprints (MACCS and ECFP).

#### 4.4.2 Gradient Boosting

Gradient boosting is another ensemble technique that builds models sequentially, with each new tree attempting to correct the errors of the preceding ensemble. This approach focuses on minimizing the overall prediction error rather than solely reducing variance,^48^ which often results in high predictive accuracy when properly regularized. We employed gradient boosting on both molecular descriptors and fingerprint features (MACCS and ECFP).

#### 4.4.3 Feedforward Neural Network

The feedforward neural network (FeedFwd) architecture consists of two main components: a numerical feature encoder and a classifier. The encoder processes the input molecular descriptors through a series of dense blocks, each composed of a fully connected layer, a normalization layer, a nonlinear activation function, and a dropout layer for regularization.^49^ The resulting feature representations are then fed into a classifier module composed of additional dense blocks and a final output layer with softmax activation for classification.^50^ This design enables the FeedFwd to effectively capture and learn the intricate, nonlinear interactions present in molecular descriptor data.

#### 4.4.4 Sparse Encoder

The sparse encoder is specifically tailored for high-dimensional, sparse input data such as MACCS and ECFP fingerprints. It begins with an index encoder that incorporates an embedding layer^51^ to transform discrete indices—representing the positions within the fingerprint vectors—into continuous dense embeddings. These embeddings capture latent structural information, which is then passed through a classifier to output a permeability probability. This model design leverages the sparse nature of fingerprint data while efficiently learning underlying chemical patterns.

#### 4.4.5 Graph Convolutional Neural Network

Graph convolutional neural networks are designed to directly process graph structures, thus capturing both local and global topological features inherent in the chemical compounds. The graph encoder module applies graph-specific convolutional operations to aggregate and transform atom and bond features, enabling the model to learn rich, structure-aware representations.^52^ Additionally, internal embedding layers are utilized to handle categorical atom features, ensuring smooth integration into the convolutional framework. The final classification module translates these learned representations into BBB permeability predictions, offering a powerful approach to modeling complex molecular interactions.

#### 4.4.6 ChemBERTa

ChemBERTa is a transformer-based chemical language model pretrained on extensive chemical data sets.^39^ In our implementation, the pretrained encoder is used to generate contextualized embeddings from SMILES token sequences. A downstream classifier—comprising a dense block and an output layer—is then fine-tuned to predict BBB permeability. To preserve the valuable pretrained knowledge, most of the encoder parameters remain frozen during fine-tuning. This strategy enables the model to leverage prior chemical language understanding while adapting to the specific BBB permeability prediction task.

### 4.5 Parameter Tuning

For our tree ensemble models, tuning focused on selecting appropriate splitting criteria and addressing the class imbalance. In random forests, the Gini impurity criterion was employed to optimize split decisions, while class weighting was integrated to counteract imbalance in the data set. Similarly, for gradient boosting, splits were determined using the mean-squared error improvement score, with class weights applied to ensure balanced learning across classes.

For our deep learning architectures, we adopted the AdamW optimizer,^53^ which incorporates weight decay directly into the update rule to enhance generalization and mitigate overfitting. The training was conducted using a mini-batch approach,^54^ which improves convergence stability and computational efficiency. To further address class imbalance and focus learning on challenging examples, we selected a class-weighted focal loss.^55^ This loss function not only downweights well-classified instances but also assigns higher importance to misclassified samples, thereby promoting a more balanced learning process. Additionally, a cosine annealing learning rate scheduler^56^ was used to dynamically adjust the learning rate during training, helping the model to navigate local minima and achieve smoother convergence. Finally, weight initialization was tailored to the activation functions used within each network component. Layers employing GELU and ReLU activations were initialized with the Kaiming (He) initialization technique,^57^ which is effective at maintaining activation variance across layers. In contrast, layers that ended with a softmax activation function were initialized using Xavier initialization,^58^ ensuring that the weights start in a balanced state conducive to stable gradient propagation.

### 4.6 Hyperparameter Tuning

For each pipeline—defined by a combination of model, input features, and training data set—a distinct set of hyperparameters was established. These included hyperparameters such as the number of trees, tree depth, class weights, learning rate, batch size, embedding dimension, number of layers, and dropout rate, adjusted according to the specific model’s requirements. A range of discrete values were proposed for each hyperparameter. For example, learning rates of 0.001, 0.0001, and 0.00001 were considered, while batch sizes of 32, 64, and 128 were explored. The hyperparameter space remained discrete to avoid potential overfitting caused by the curse of dimensionality.^59^

Hyperparameter optimization was performed using a sequential Bayesian optimization approach based on tree-structured Parzen estimators.^60^ This iterative process involved training an auxiliary probabilistic generative model to achieve two key objectives: 1) estimate the probability of achieving the target performance metric—macro F1-score—for a given set of hyperparameters and 2) propose new hyperparameter values at each iteration to improve the performance metric.

Each proposed set of hyperparameters was evaluated by training the model on four K-folds of the training data. The macro-F1 score used for evaluation was the average of the individual macro-F1 scores obtained from the validation sets of these folds. Notably, the test set data were excluded from the hyperparameter tuning process.

Once the *optimal* hyperparameters were obtained, retraining was carried out for the full training sets for the random, exact, and ExApr splits.

### 4.7 Evaluation

#### 4.7.1 Performance Inflation from Overrepresentation Bias

To evaluate the impact of overrepresentation bias, we assessed the performance of each pipeline, defined by a unique combination of model, input features, and training data set. The evaluation was conducted on three distinct test sets: random, exact, and ExApr.

Performance was quantified using two standard metrics: the area under the receiver operating characteristic curve (AUC) and the macro F1-score. To specifically measure the performance overestimation caused by near-duplicate compounds in the test data, we introduced a custom metric termed *Performance inflation* (Δ). This metric quantifies the relative performance increase observed on the random test set (an optimistic estimate containing near-duplicates) compared to the ExApr test set (a conservative estimate without near-duplicate compounds). It is defined as

14 $$\Delta M=\frac{M_{Random}-M_{ExApr}}{M_{ExApr}}$$

where *M* is a given performance metric (e.g., AUC or F1-Score) and *M*_Random_ and *M*_ExApr_ are its values on the corresponding test sets.

To assess the stability and statistical significance of our results, we computed 95% confidence intervals for all reported metrics using a nonparametric bootstrap procedure with 1000 resamples.^61^ For the performance inflation metric, the confidence interval was derived from the bootstrapped distributions of the base metrics. Let **M**_Random_ = {*m*_Random,1_, ..., *m*_Random,*N*_} and **M**_ExApr_ = {*m*_ExApr,1_, ..., *m*_ExApr,*N*_} be the distributions of a metric obtained from *N* = 1000 bootstrap samples on the Random and ExApr test sets, respectively. The confidence interval was then computed as follows:

1. Distribution of performance inflation: a distribution for performance inflation, **Δ**, was generated by applying the formula element-wise to the corresponding pairs of bootstrapped values:

15 $$\Delta =\frac{M_{Random}-M_{ExApr}}{M_{ExApr}}$$

2. Percentile Calculation: the 95% confidence interval was determined by the 2.5th and 97.5th percentiles of the resulting Δ distribution.

#### 4.7.2 Disentangling In-Distribution vs Out-Of-Distribution Evaluation

A comprehensive evaluation of machine learning models in chemoinformatics—such as BBBP—requires a clear methodological distinction between Out-distribution (OD) extrapolation and In-distribution (ID) interpolation. These two paradigms measure fundamentally different aspects of model utility, corresponding to distinct phases of the drug discovery pipeline.

To asses OD extrapolation, Scaffold splitting^23^ is widely categorized as the gold standard. By forcing a structural domain shift between the training and test sets, it assesses a model’s ability to extrapolate to entirely novel chemotypes. This capability is critical for early-stage virtual screening campaigns aiming to discover new active scaffolds.

Hence, we implemented a stratified scaffold split strategy to evaluate the OD performance. We clustered the compounds based on their core Bemis-Murcko topological skeletons.^62^ We then partitioned the data set such that the scaffolds assigned to the test set were completely disjointed from those present in the training set. This partitioning approach forces a hard topological domain shift, effectively eliminating the possibility of the model succeeding artificially through structural familiarity or feature leakage. As with all evaluation partitions in our methodology, we ensured that this scaffold split remained strictly stratified to maintain consistent permeability class proportions between the training and test sets.

However, relying exclusively on OD evaluation ignores an important reality of pharmacological development. Much of real-world drug discovery—specifically the lead optimization phase—relies on ID interpolation. During lead optimization, medicinal chemists work within a populated chemical space (a congeneric series), making minor structural modifications to a core active scaffold to optimize properties like BBBP while minimizing off-target toxicity. Evaluating a model’s utility for this specific task using a scaffold split is mathematically misaligned, as it forces an artificial domain shift that disrupts the local chemical neighborhood the model needs to interpolate within.

To construct this ID split, we considered the previously derived Bemis-Murcko topological skeletons. We generated an *In-distribution Random* test set by enforcing a strict inclusion criterion: all scaffolds present in this test set were required to already be represented within the training set. This guarantees that no major topological domain shift occurs between training and evaluation, isolating the model’s ability to interpolate within a familiar domain.

However, as our study highlights, standard random sampling within a shared scaffold space is highly susceptible to overrepresentation bias. Models can easily exploit feature leakage, memorizing structurally redundant analogs rather than learning true structure–property relationships. Therefore, to measure true *hard interpolation* without the artificial performance inflation caused by near-duplicate memorization, we applied our automated filtering framework to this baseline. By removing all instances from the test set that shared exact or approximate feature collisions with the training data, we created the sanitized *In-distribution ExApr* test set and evaluated the models’ performance on this refined split.

## 5 Results

### 5.1 Automatic Detection of Overrepresentation Bias

Figure 3 shows the results of applying the proposed automatic threshold extraction method to determine when two distinct compounds can be categorized as approximate collisions—and thus as near-duplicate compounds.

**Figure 3:**
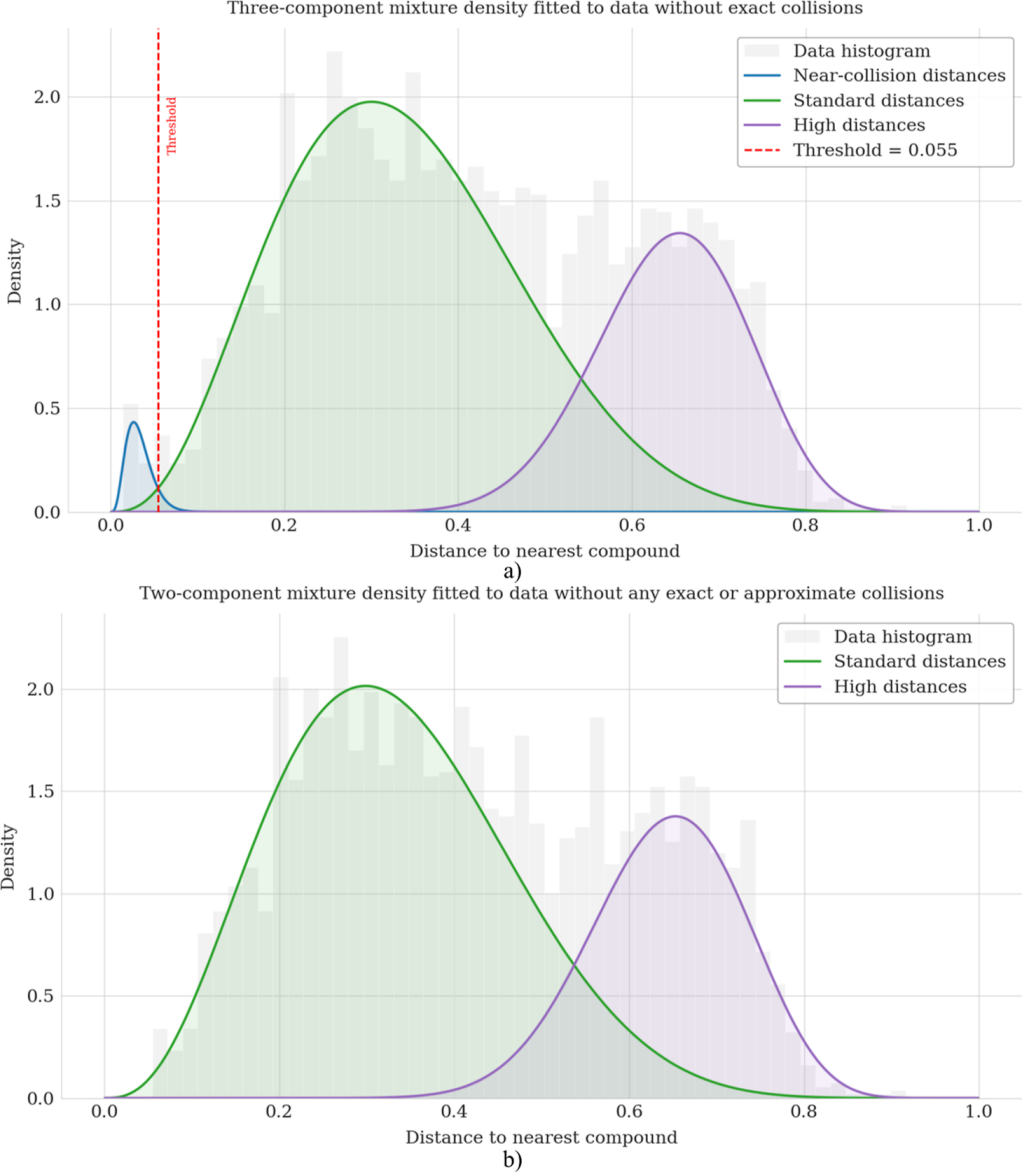
Automatic detection of overrepresentation bias from approximate feature collisions. The latent distributions for the model with the lowest Bayesian information criterion are shown for (a) the exact train set and (b) the exact and approximate train set. In panel (a), the threshold is extracted automatically at the intersection of the near-duplicates and standard nearest-distance probability density functions. In panel (b), compounds whose nearest distances are equal or fall below this threshold have been filtered out. In both panels, light-gray bars depict the density histogram of all nearest distances, and the colored curves represent the individual mixture components.

From Figure 3a, we infer that the three-component beta mixture model—the model with the lowest BIC in the exact train set—provides an excellent fit to the underlying nearest-distance data. Each component distribution is well-defined, allowing us to extract a clear threshold at the intersection of the near-collisions and standard distance distributions, which occurs at approximately 0.055.

After applying this automatically calculated threshold to the exact training set, the two-component beta mixture model—comprising a standard distance component and a higher distance component—emerged as the best fit (lowest BIC). As shown in Figure 3b, filtering out compounds whose nearest distances are equal or fall below the threshold removes the near-collisions, and the resulting mixture accurately approximates the remaining distribution.

To assess the robustness of the automatically derived threshold and ensure that it was not an artifact of a specific data partition, we conducted a sensitivity analysis. We repeated the full splitting and threshold detection pipeline 30 times using different random seeds for the initial training and test stratification. The derived threshold exhibited high stability across all iterations, yielding a mean value of 0.054 with a standard deviation of 0.003. This low variance confirms that the threshold is driven by the intrinsic structure of the chemical data set rather than stochastic variations in data sampling.

Furthermore, this analysis highlights the limitation of using standard heuristics (e.g., Jaccard distance < 0.1). In our data set, a fixed threshold of 0.1 would be located significantly to the right of the optimal intersection point (0.055), encroaching upon the distribution of standard distances (Figure 3a). This would result in the erroneous removal of distinct, informative compounds, whereas our probabilistic approach minimizes the Bayes’ classification error between near-duplicates and distinct entities.

### 5.2 Overrepresentation Bias Visualization

Figure 4 visualizes the effects of overrepresentation bias using two-dimensional multidimensional scaling (MDS) projections of the test set compounds. The embeddings were generated for each feature representation—molecular descriptors, molecular access system (MACCS) fingerprints, circular fingerprints (ECFP), graph features, and SMILES tokens—across the three test sets (random, exact, and ExApr). Each point represents a compound, colored by its blood-brain barrier (BBB) permeability label (blue for nonpermeable, red for permeable). This visualization reveals how overrepresentation bias influences the distribution of compounds within the feature space.

**Figure 4:**
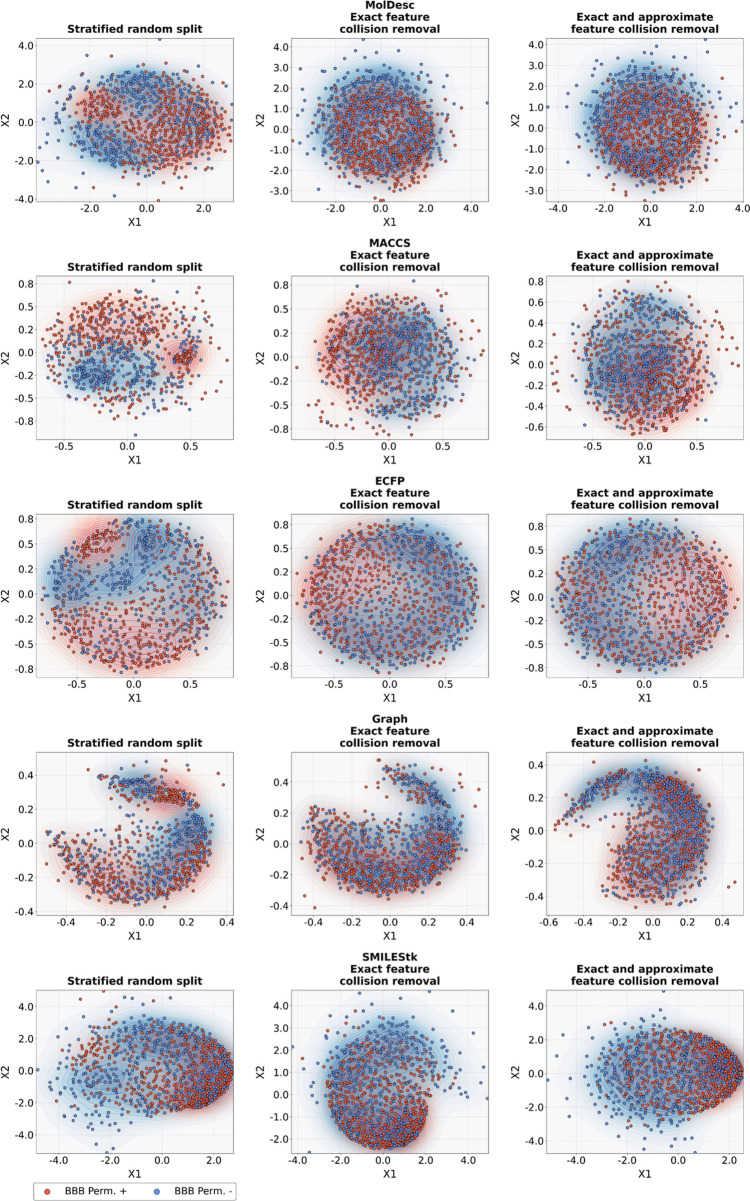
Two-dimensional multidimensional scaling embeddings of test set compounds, based on pairwise compound distances, for each feature and data set. Abbreviations: MolDesc., molecular descriptors; MACCS, molecular access system fingerprints; ECFP, extended-circular fingerprints; Graph, graph features; SMLILEStk, tokens from the simplified molecular input line entry system; BBB Perm., blood-brain barrier permeability.

Although the test sets for each split (random, exact, and ExApr) contain the same number of compounds and identical class frequencies, their spatial distributions differ markedly.

In the random splits, compounds with the same permeability label form distinct clusters, creating dense monochromatic regions. This pattern, consistent across all feature types, indicates that random-based partitioning fails to uniformly sample the chemical space. Consequently, these test sets overrepresent specific feature space regions, which can lead to an inflated performance estimates.

Conversely, the exact and ExApr splits exhibit a more homogeneous distribution. The ExApr split, in particular, shows a diffuse mixing of both permeable and nonpermeable compounds. This suggests a more comprehensive sampling of the chemical space, which mitigates bias by preventing the formation of dense single-class clusters and ensuring a more representative evaluation.

Figure 5 provides a visual confirmation of the overrepresentation bias phenomenon using MDS embeddings of the ECFP feature space. The four panels contrast the spatial densities of the test set compounds under different partitioning strategies, revealing how overrepresentation bias physically manifests within the model’s coordinate system.

**Figure 5:**
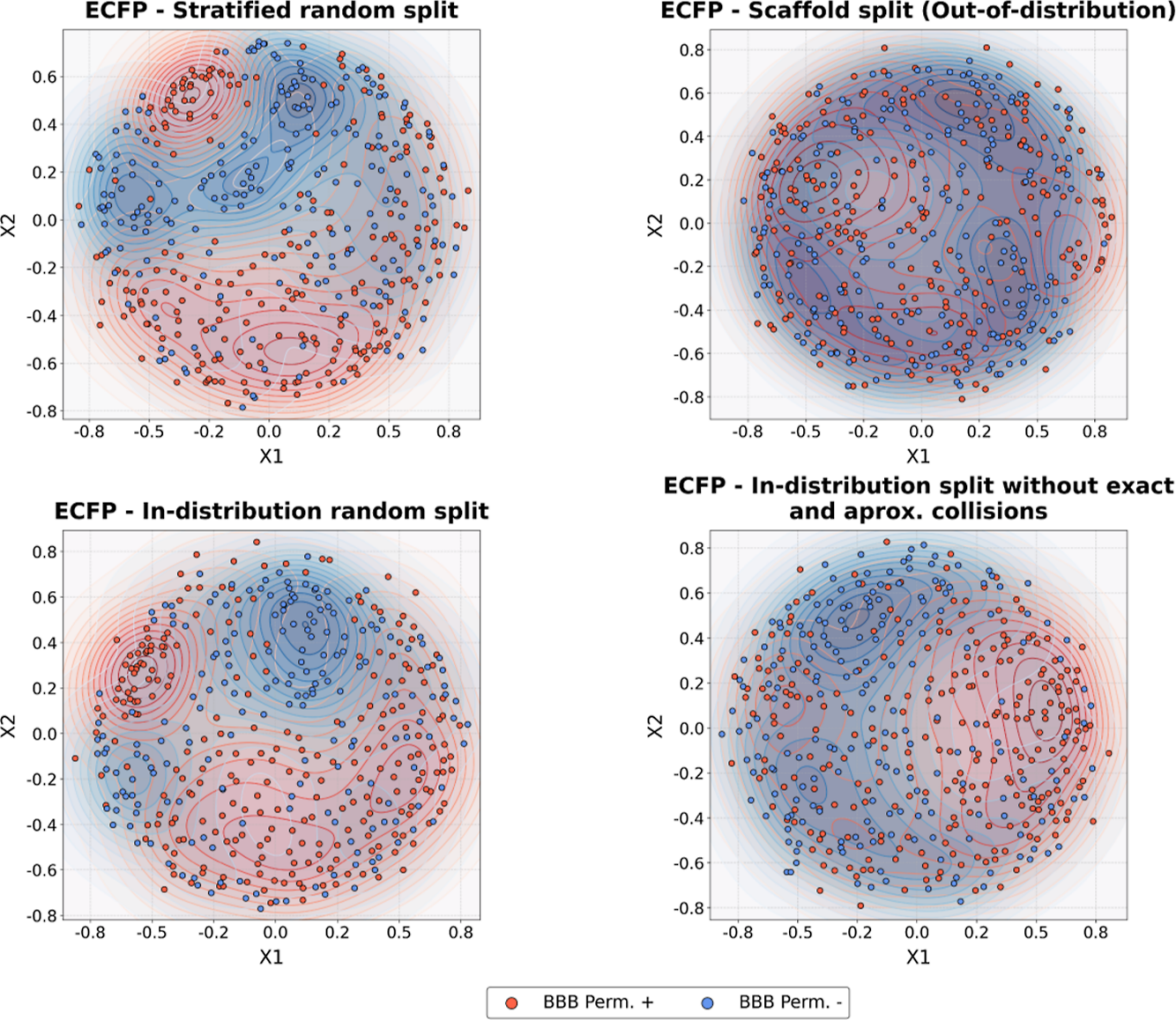
Two-dimensional multidimensional scaling embeddings of test set compounds, based on pairwise compound distances, considering different partition strategies. Abbreviations: MolDesc., molecular descriptors; MACCS, molecular access system fingerprints; ECFP, extended-circular fingerprints; Graph, graph features; SMLILEStk, tokens from the simplified molecular input line entry system; Aprox., approximate; BBB Perm., blood-brain barrier permeability.

In both the standard *stratified random split* (top left) and the *in-distribution random split* (bottom left), the feature space is dominated by tightly localized, high-density clusters of compounds sharing identical permeability labels. These dense, monochromatic regions (highlighted by the concentrated contour rings) are the direct visual representations of overrepresentation bias. They demonstrate that standard random sampling fails to uniformly partition the chemical space, instead populating the test set with near duplicates that allow the model to achieve artificially high performance through *easy interpolation* (memorization).

Conversely, the evaluation paradigms on the right-hand side of the figure actively disrupt these artifacts. The *scaffold split* (top right) forces a hard structural separation, which visually diffuses the data and eliminates the localized clusters. However, this represents an extreme out-of-distribution (extrapolation) shift.

A critical insight is provided by the *in-distribution split without exact and approximate collisions* (bottom right). When the automated filter is applied to the in-distribution space, the highly concentrated, biased clusters seen in the left panels are systematically dispersed. The resulting distribution is markedly more uniform, smoothly covering the chemical domain without excessively emphasizing redundant structural motifs while remaining in distribution. This visual evidence confirms that the ExApr filtering successfully sanitizes the interpolation space, removing the feature collisions that drive data leakage while preserving the native boundaries required for unbiased *hard interpolation* evaluation.

### 5.3 Performance Estimation

Next, we present the AUC and F1-score macro-averaged, calculated for each pipeline developed for the random, exact, and ExApr test sets. Note that additional performance metrics can be found in the Supporting Information.

#### 5.3.1 Area under Curve

Table 3 presents the AUC values, with 95% nonparametric confidence intervals shown in brackets, and the percentage performance inflation (Δ %) for each pipeline.

**Table 3:** Area under Curve (AUC), with 95% Nonparametric Confidence Intervals Shown in Brackets, and the Percentage Performance Inflation (Δ %) for Each Developed Pipeline^a^

inputs	model	train set	test set			Δ %
			random	exact	ExApr	
MolDesc	RandFor	random	0.958 [0.945, 0.97]	0.856 [0.828, 0.878]	0.855 [0.829, 0.88]	12.00 [7.40, 17.00]
MolDesc	RandFor	exact	0.954 [0.941, 0.967]	0.854 [0.828, 0.878]	0.853 [0.827, 0.878]	11.80 [7.20, 16.90]
MolDesc	RandFor	ExaApr	0.955 [0.943, 0.968]	0.856 [0.83, 0.88]	0.856 [0.829, 0.88]	11.60 [7.20, 16.80]
MolDesc	GradBst	random	0.958 [0.945, 0.969]	0.865 [0.84, 0.887]	0.864 [0.838, 0.888]	10.90 [6.40, 15.60]
MolDesc	GradBst	exact	0.957 [0.944, 0.969]	0.866 [0.841, 0.89]	0.865 [0.84, 0.889]	10.60 [6.20, 15.40]
MolDesc	GradBst	ExaApr	0.956 [0.942, 0.968]	0.868 [0.843, 0.891]	0.867 [0.842, 0.892]	10.30 [5.60, 15.00]
MolDesc	FeedFwd	random	0.897 [0.875, 0.918]	0.79 [0.759, 0.821]	0.791 [0.759, 0.822]	13.40 [6.40, 20.90]
MolDesc	FeedFwd	exact	0.885 [0.861, 0.907]	0.788 [0.757, 0.817]	0.788 [0.756, 0.819]	12.30 [5.10, 20.00]
MolDesc	FeedFwd	ExaApr	0.886 [0.863, 0.908]	0.786 [0.754, 0.815]	0.786 [0.754, 0.818]	12.70 [5.50, 20.40]
MACCS	RandFor	random	0.954 [0.941, 0.966]	0.848 [0.821, 0.874]	0.847 [0.822, 0.874]	14.70 [9.30, 20.30]
MACCS	RandFor	exact	0.951 [0.938, 0.963]	0.847 [0.82, 0.872]	0.847 [0.822, 0.872]	14.30 [8.90, 20.10]
MACCS	RandFor	ExaApr	0.951 [0.938, 0.964]	0.85 [0.823, 0.875]	0.849 [0.824, 0.875]	15.00 [9.40, 20.70]
MACCS	GradBst	random	0.947 [0.932, 0.962]	0.837 [0.809, 0.863]	0.837 [0.81, 0.865]	14.10 [9.20, 19.90]
MACCS	GradBst	exact	0.949 [0.935, 0.962]	0.842 [0.816, 0.868]	0.842 [0.816, 0.868]	15.10 [9.70, 21.10]
MACCS	GradBst	ExaApr	0.947 [0.932, 0.961]	0.841 [0.813, 0.867]	0.842 [0.817, 0.868]	14.30 [9.10, 20.10]
MACCS	SparEnc	random	0.923 [0.903, 0.941]	0.822 [0.792, 0.85]	0.822 [0.796, 0.848]	16.20 [10.00, 23.60]
MACCS	SparEnc	exact	0.937 [0.919, 0.952]	0.833 [0.805, 0.859]	0.831 [0.806, 0.859]	17.10 [10.40, 23.90]
MACCS	SparEnc	ExaApr	0.94 [0.924, 0.954]	0.819 [0.79, 0.847]	0.819 [0.793, 0.847]	16.80 [10.00, 24.10]
ECFP	RandFor	random	0.939 [0.926, 0.953]	0.819 [0.788, 0.845]	0.819 [0.792, 0.847]	12.60 [7.70, 17.50]
ECFP	RandFor	exact	0.935 [0.921, 0.95]	0.818 [0.788, 0.844]	0.818 [0.791, 0.846]	12.30 [7.60, 17.20]
ECFP	RandFor	ExaApr	0.935 [0.92, 0.95]	0.814 [0.783, 0.841]	0.813 [0.787, 0.841]	12.00 [7.20, 17.00]
ECFP	GradBst	random	0.952 [0.939, 0.966]	0.834 [0.805, 0.861]	0.834 [0.806, 0.86]	13.10 [7.70, 18.80]
ECFP	GradBst	exact	0.951 [0.937, 0.964]	0.828 [0.8, 0.855]	0.826 [0.796, 0.854]	12.70 [7.70, 17.90]
ECFP	GradBst	ExaApr	0.95 [0.937, 0.963]	0.832 [0.804, 0.86]	0.831 [0.802, 0.859]	12.50 [7.40, 17.60]
ECFP	SparEnc	random	0.924 [0.905, 0.942]	0.798 [0.765, 0.827]	0.795 [0.762, 0.823]	12.30 [6.50, 18.20]
ECFP	SparEnc	exact	0.932 [0.914, 0.949]	0.797 [0.765, 0.826]	0.796 [0.766, 0.828]	12.80 [7.00, 18.10]
ECFP	SparEnc	ExaApr	0.924 [0.904, 0.943]	0.791 [0.758, 0.821]	0.791 [0.76, 0.822]	14.80 [9.10, 20.30]
Graph	GraConv	random	0.936 [0.918, 0.952]	0.819 [0.791, 0.848]	0.818 [0.789, 0.846]	14.40 [8.50, 20.70]
Graph	GraConv	exact	0.922 [0.904, 0.939]	0.799 [0.769, 0.829]	0.797 [0.767, 0.827]	15.70 [9.30, 22.40]
Graph	GraConv	ExaApr	0.924 [0.904, 0.941]	0.795 [0.767, 0.829]	0.793 [0.764, 0.824]	16.50 [9.70, 23.20]
SMILEStk	ChBERTa	random	0.933 [0.916, 0.949]	0.831 [0.805, 0.857]	0.83 [0.802, 0.857]	12.40 [6.90, 18.30]
SMILEStk	ChBERTa	exact	0.918 [0.898, 0.936]	0.847 [0.82, 0.874]	0.844 [0.819, 0.87]	8.80 [3.20, 14.30]
SMILEStk	ChBERTa	ExaApr	0.917 [0.896, 0.937]	0.849 [0.82, 0.873]	0.846 [0.819, 0.872]	8.40 [2.80, 14.40]

a Abbreviations: MolDesc., molecular descriptors; MACCS, molecular access system fingerprints; ECFP, extended-circular fingerprints; Graph, graph features; SMLILEStk, tokens from the simplified molecular input line entry system; RandFor, random forest; GradBst, gradient boosting; FeedFwd, feed forward deep neural network; SparEnc, sparse encoder; GraConv, graph convolutional network; ChBERTa, chemical bidirectional encoder representations from transformers; ExApr, exact and approximate.

As shown in Table 3, the highest AUC values are consistently achieved when models are evaluated on the random test set, while the lowest values occur on the ExApr test set. This performance gap is substantial, with performance inflation values often exceeding 10%. Crucially, the 95% confidence interval for this inflation metric never includes zero, confirming that the drop in the performance is statistically significant.

Interestingly, these differences among test sets do not appear in the training sets; models trained on the random, exact, or ExApr data sets using the same features do not show such clear variations.

In terms of model performance, decision tree models outperform their deep learning counterparts for the comparable feature types—namely, molecular descriptors, MACCS and ECFP fingerprints—in the random, exact, and ExApr test sets. Notably, for the random test set, the best-performing pipelines are the random forest and gradient boosting models using molecular descriptors as input features, trained on the random data set. For the exact test set, the highest AUC is achieved by the gradient boosting model with molecular descriptor features, trained on the ExApr data set. Finally, for the ExApr test set, the top-performing pipeline is once again the gradient boosting model with molecular descriptor inputs, trained on the ExApr data set.

Table 4 illustrates the dichotomy between interpolation and extrapolation paradigms as well as the profound impact of overrepresentation bias on model evaluation. Across all input features and architectures, a clear and consistent performance hierarchy emerges: In-distribution Random > In-distribution ExApr > Out-distribution. Models evaluated on the raw In-distribution random test set yield highly optimistic AUC metrics, frequently exceeding 0.95. However, when the ExApr filter is applied to remove exact and approximate feature collisions (in-distribution ExApr), performance drops significantly to a more rigorous estimate of true in-domain interpolation (ranging from 0.770 to 0.857). The lack of overlap in the 95% confidence intervals between these two in-distribution sets suggests that the near-perfect metrics observed in standard random splits are largely artifacts of data leakage and trivial memorization.

**Table 4:** Area under Curve (AUC) Comparing In-Distribution and Out-Of-Distribution Test Sets, with 95% Nonparametric Confidence Intervals Shown in Brackets^a^

inputs	model	test set		
		In-dist. Random	In-dist. ExApr	Out-dist.
MolDesc	RandFor	0.965 [0.948, 0.979]	0.849 [0.816, 0.882]	0.818 [0.796, 0.839]
MolDesc	GradBst	0.967 [0.952, 0.98]	0.857 [0.827, 0.89]	0.809 [0.787, 0.83]
MolDesc	FeedFwd	0.897 [0.867, 0.923]	0.77 [0.727, 0.816]	0.758 [0.736, 0.781]
MACCS	RandFor	0.966 [0.949, 0.98]	0.853 [0.821, 0.886]	0.806 [0.784, 0.827]
MACCS	GradBst	0.962 [0.945, 0.977]	0.836 [0.804, 0.873]	0.789 [0.765, 0.809]
MACCS	SparEnc	0.945 [0.924, 0.966]	0.827 [0.791, 0.866]	0.772 [0.746, 0.794]
ECFP	RandFor	0.952 [0.934, 0.969]	0.816 [0.782, 0.854]	0.757 [0.732, 0.78]
ECFP	GradBst	0.965 [0.949, 0.979]	0.842 [0.81, 0.876]	0.769 [0.746, 0.791]
ECFP	SparEnc	0.951 [0.928, 0.97]	0.809 [0.772, 0.851]	0.699 [0.672, 0.725]
Graph	GraConv	0.952 [0.932, 0.969]	0.823 [0.785, 0.862]	0.729 [0.703, 0.752]
SMILEStk	ChBERTa	0.947 [0.929, 0.965]	0.831 [0.794, 0.871]	0.778 [0.754, 0.801]

a Abbreviations: MolDesc., molecular descriptors; MACCS, molecular access system fingerprints; ECFP, extended-circular fingerprints; Graph, graph features; SMLILEStk, tokens from the simplified molecular input line entry system; RandFor, random forest; GradBst, gradient boosting; FeedFwd, feed forward deep neural network; SparEnc, sparse encoder; GraConv, graph convolutional network; ChBERTa, chemical bidirectional encoder representations from transformers; Dist, distribution; ExApr, exact and approximate.

Furthermore, the out-distribution (scaffold split) evaluation predictably yields the lowest AUC values (ranging from 0.699 to 0.818), reflecting the inherent difficulty of extrapolating to entirely novel chemotypes. Regarding specific architectures, traditional tree-based models (gradient boosting and random forest) generally demonstrate a greater resilience than deep learning approaches across both the sanitized interpolation (ExApr) and extrapolation (Out-distribution) tasks. Specifically, the random forest model utilizing molecular descriptors achieved the highest extrapolation performance.

#### 5.3.2 F1-Score Macro

Table 5 presents the F1-score macro (average of individual F1-scores per class) values, with 95% nonparametric confidence intervals shown in brackets, and the percentage performance inflation (Δ%) for each pipeline.

**Table 5:** F1-Score Macroaveraged, with 95% Nonparametric Confidence Intervals Shown in Brackets, and the Percentage Performance Inflation (Δ %) for Each Developed Pipeline^a^

inputs	model	train set	test set			Δ %
			random	exact	ExApr	
MolDesc	RandFor	Random	0.88 [0.858, 0.902]	0.764 [0.734, 0.793]	0.763 [0.733, 0.791]	15.30 [8.50, 23.10]
MolDesc	RandFor	Exact	0.879 [0.858, 0.901]	0.764 [0.734, 0.793]	0.763 [0.732, 0.792]	15.20 [8.30, 23.10]
MolDesc	RandFor	ExaApr	0.878 [0.856, 0.899]	0.764 [0.732, 0.791]	0.764 [0.735, 0.793]	14.90 [7.90, 22.30]
MolDesc	GradBst	Random	0.901 [0.881, 0.921]	0.789 [0.761, 0.815]	0.787 [0.757, 0.814]	14.50 [8.20, 21.70]
MolDesc	GradBst	Exact	0.901 [0.88, 0.921]	0.781 [0.751, 0.81]	0.781 [0.753, 0.808]	15.40 [8.90, 22.30]
MolDesc	GradBst	ExaApr	0.896 [0.874, 0.916]	0.77 [0.74, 0.798]	0.77 [0.741, 0.798]	16.40 [9.50, 23.60]
MolDesc	FeedFwd	Random	0.826 [0.8, 0.85]	0.718 [0.686, 0.749]	0.717 [0.686, 0.747]	15.20 [7.10, 23.90]
MolDesc	FeedFwd	Exact	0.813 [0.785, 0.838]	0.717 [0.685, 0.746]	0.717 [0.685, 0.749]	13.40 [4.80, 22.30]
MolDesc	FeedFwd	ExaApr	0.807 [0.778, 0.833]	0.717 [0.685, 0.747]	0.717 [0.685, 0.748]	12.60 [4.00, 21.60]
MACCS	RandFor	Random	0.888 [0.867, 0.91]	0.767 [0.739, 0.796]	0.767 [0.739, 0.796]	18.80 [11.00, 27.20]
MACCS	RandFor	Exact	0.89 [0.869, 0.91]	0.772 [0.743, 0.802]	0.772 [0.745, 0.801]	15.40 [7.40, 23.80]
MACCS	RandFor	ExaApr	0.882 [0.861, 0.903]	0.77 [0.742, 0.798]	0.77 [0.742, 0.8]	16.50 [8.60, 24.90]
MACCS	GradBst	Random	0.885 [0.862, 0.905]	0.772 [0.742, 0.8]	0.772 [0.745, 0.802]	18.60 [11.20, 26.40]
MACCS	GradBst	Exact	0.895 [0.874, 0.914]	0.77 [0.743, 0.798]	0.769 [0.741, 0.797]	17.20 [10.10, 24.70]
MACCS	GradBst	ExaApr	0.892 [0.871, 0.911]	0.769 [0.739, 0.797]	0.77 [0.742, 0.799]	17.50 [10.50, 25.10]
MACCS	SparEnc	Random	0.882 [0.861, 0.903]	0.755 [0.723, 0.783]	0.756 [0.728, 0.784]	19.70 [12.20, 28.60]
MACCS	SparEnc	Exact	0.88 [0.857, 0.9]	0.756 [0.726, 0.784]	0.754 [0.724, 0.784]	19.40 [11.30, 27.80]
MACCS	SparEnc	ExaApr	0.88 [0.858, 0.901]	0.734 [0.704, 0.764]	0.734 [0.706, 0.764]	22.20 [14.20, 30.80]
ECFP	RandFor	Random	0.858 [0.836, 0.88]	0.722 [0.688, 0.751]	0.722 [0.692, 0.753]	15.80 [8.90, 23.10]
ECFP	RandFor	Exact	0.834 [0.809, 0.858]	0.723 [0.69, 0.755]	0.723 [0.693, 0.753]	15.30 [8.50, 22.10]
ECFP	RandFor	ExaApr	0.835 [0.811, 0.858]	0.716 [0.682, 0.746]	0.717 [0.687, 0.747]	14.50 [7.60, 21.70]
ECFP	GradBst	Random	0.881 [0.861, 0.901]	0.743 [0.712, 0.774]	0.743 [0.713, 0.774]	14.60 [7.50, 21.50]
ECFP	GradBst	Exact	0.878 [0.858, 0.898]	0.749 [0.718, 0.78]	0.749 [0.72, 0.779]	16.40 [9.70, 23.30]
ECFP	GradBst	ExaApr	0.881 [0.861, 0.901]	0.75 [0.72, 0.78]	0.75 [0.72, 0.779]	15.80 [9.00, 22.80]
ECFP	SparEnc	Random	0.87 [0.848, 0.894]	0.729 [0.696, 0.758]	0.727 [0.695, 0.756]	16.70 [9.80, 24.00]
ECFP	SparEnc	Exact	0.869 [0.847, 0.892]	0.731 [0.7, 0.761]	0.728 [0.698, 0.761]	16.70 [9.30, 24.30]
ECFP	SparEnc	ExaApr	0.87 [0.847, 0.892]	0.712 [0.68, 0.741]	0.712 [0.682, 0.742]	19.90 [12.30, 27.60]
Graph	GraConv	Random	0.882 [0.861, 0.903]	0.753 [0.723, 0.783]	0.753 [0.724, 0.783]	17.10 [10.00, 24.70]
Graph	GraConv	Exact	0.868 [0.845, 0.889]	0.729 [0.695, 0.76]	0.727 [0.698, 0.759]	19.40 [11.30, 27.40]
Graph	GraConv	ExaApr	0.867 [0.846, 0.888]	0.731 [0.699, 0.762]	0.73 [0.698, 0.76]	18.80 [11.30, 27.20]
SMILEStk	ChBERTa	Random	0.875 [0.852, 0.897]	0.764 [0.732, 0.792]	0.764 [0.736, 0.791]	14.50 [7.70, 21.90]
SMILEStk	ChBERTa	Exact	0.835 [0.807, 0.859]	0.756 [0.727, 0.784]	0.754 [0.724, 0.784]	10.70 [2.90, 18.60]
SMILEStk	ChBERTa	ExaApr	0.858 [0.833, 0.882]	0.772 [0.742, 0.801]	0.771 [0.741, 0.799]	11.30 [4.30, 19.00]

a Abbreviations: MolDesc., molecular descriptors; MACCS, molecular access system fingerprints; ECFP, extended-circular fingerprints; Graph, graph features; SMLILEStk, tokens from the simplified molecular input line entry system; RandFor, random forest; GradBst, gradient boosting; FeedFwd, feed forward deep neural network; SparEnc, sparse encoder; GraConv, graph convolutional network; ChBERTa, chemical bidirectional encoder representations from transformers; ExApr, exact and approximate.

Table 5 reveals a consistent pattern across all models and input features: the highest F1 scores were achieved on the random test set and the lowest on the ExApr test set. This performance gap is wide with inflation values frequently surpassing 15%. Notably, the 95% confidence interval for this inflation metric never includes zero, confirming that the drop in the performance is statistically significant. This trend aligns with the previous results for the AUC metric, as shown in Table 3.

Moreover, these differences between the test sets are not present in the training sets. No clear differences are observed when comparing models trained with the random, exact, or ExApr data sets using the same features and models.

Regarding model performance, decision tree-based models clearly outperform their deep learning counterparts when using descriptor features, and to a lesser extent when using fingerprint features (MACCS and ECFP).

For the random test set, the gradient boosting model trained on the random data set with molecular descriptors as input features achieved the best performance. The same configuration also attained the highest F1 score on the exact test set. Finally, for the ExApr test set, the gradient boosting model using molecular descriptors trained on the random data set again outperformed all others.

Table 6 reinforces the conclusions drawn from the AUC analysis, demonstrating the impact of overrepresentation bias on the macro-F1 score. The results mirror the previously established hierarchy, with the In-Distribution Random test set yielding drastically inflated metrics, frequently approaching or exceeding 0.90.

**Table 6:** F1-Score Macroaveraged Comparing In-Distribution and Out-Of-Distribution Test Sets, with 95% Nonparametric Confidence Intervals Shown in Brackets^a^

inputs	model	test set		
		In-dist. Random	In-dist. ExApr	Out-dist.
MolDesc	RandFor	0.882 [0.85, 0.911]	0.747 [0.706, 0.786]	0.672 [0.646, 0.695]
MolDesc	GradBst	0.889 [0.86, 0.917]	0.74 [0.702, 0.782]	0.683 [0.66, 0.707]
MolDesc	FeedFwd	0.794 [0.756, 0.828]	0.706 [0.665, 0.753]	0.683 [0.658, 0.705]
MACCS	RandFor	0.892 [0.861, 0.919]	0.747 [0.708, 0.79]	0.658 [0.634, 0.679]
MACCS	GradBst	0.903 [0.876, 0.93]	0.77 [0.732, 0.81]	0.688 [0.663, 0.71]
MACCS	SparEnc	0.897 [0.867, 0.924]	0.749 [0.713, 0.788]	0.683 [0.659, 0.706]
ECFP	RandFor	0.881 [0.853, 0.912]	0.702 [0.66, 0.744]	0.567 [0.539, 0.593]
ECFP	GradBst	0.896 [0.867, 0.922]	0.759 [0.722, 0.798]	0.644 [0.618, 0.67]
ECFP	SparEnc	0.906 [0.877, 0.931]	0.75 [0.71, 0.788]	0.624 [0.6, 0.647]
Graph	GraConv	0.899 [0.872, 0.925]	0.748 [0.709, 0.787]	0.657 [0.633, 0.682]
SMILEStk	ChBERTa	0.867 [0.836, 0.899]	0.773 [0.734, 0.813]	0.697 [0.674, 0.721]

a Abbreviations: MolDesc., molecular descriptors; MACCS, molecular access system fingerprints; ECFP, extended-circular fingerprints; Graph, graph features; SMLILEStk, tokens from the simplified molecular input line entry system; RandFor, random forest; GradBst, gradient boosting; FeedFwd, feed forward deep neural network; SparEnc, sparse encoder; GraConv, graph convolutional network; ChBERTa, chemical bidirectional encoder representations from transformers; Dist, distribution; ExApr, exact and approximate.

When the data set is sanitized of exact and approximate feature collisions (In-distribution ExApr), the macro F1-scores drop into the 0.702–0.773 range. The complete lack of overlap between the 95% confidence intervals of the Random and ExApr splits across all models suggests that the optimistic F1-scores reported on standard random partitions are heavily driven by the trivial classification of near-duplicate compounds.

Furthermore, the Out-distribution (Scaffold) evaluation exposes the limitations of current architectures when forced to extrapolate, with macro F1-scores plummeting to the 0.567–0.697 range. Notably, while ensemble tree-based methods exhibited high AUC values, the transformer-based ChBERTa model utilizing 1D SMILES tokens demonstrates superior class-balanced extrapolation, achieving the highest macro F1-scores in both the rigorous interpolation (ExApr, 0.773) and extrapolation (Out-distribution, 0.697) evaluation paradigms.

## 6 Discussion

### 6.1 Performance Inflation from Overrepresentation Bias

The results presented in the previous section reveal significant differences, for both metrics considered (AUC and F1-score macro), depending on the test set used to assess the model performance. The same model exhibits far greater variability across the random, exact, and ExApr test sets than completely different models using entirely different features. These differences are particularly pronounced between the random and exact test sets, where the 95% confidence intervals do not overlap for any model compared to the smaller differences observed between the exact and ExApr test sets. This discrepancy could be explained by the fact that the number of exact collisions identified in our study is substantially higher than the number of approximate collisions. Additionally, the most substantial performance differences are found between the random and the ExApr test sets. All of this indicates that overrepresentation bias is a critical issue when it comes to accurately estimating the real model performance.

If we relate the performance metrics to the visualization of compound embeddings from the previous section, it becomes clear why models perform better on the random test set. Within these set, regions of high density containing compounds with identical label values are more readily identifiable. In contrast, in the exact test set—and especially in the ExApr test set—the compounds are more uniformly distributed, covering the chemical space in a more regular manner without excessively emphasizing any particular regions.

### 6.2 Disentangling In-Distribution vs Out-Of-Distribution Evaluation

As demonstrated in our comparative evaluations (Tables 4 and 6), OD evaluation yields the lowest performance metrics across all models and feature types compared with ID evaluation. This sharp decline accurately reflects the inherent difficulty of out-of-domain extrapolation and confirms the aggressive nature of the scaffold partitioning.

Evaluating the ID predictive capacity, however, presents its own profound methodological challenges. Standard random splitting strategies aim to preserve this in-domain space but frequently fail to distinguish between true, generalizable structure–property learning (*hard interpolation*) and the trivial memorization of near duplicates (*easy interpolation*). As our empirical results show, models evaluated on raw ID random splits yield highly optimistic, inflated metrics, frequently achieving AUC values above 0.95 and macro F1-scores approaching 0.90 (Tables 4 and 6).

The ExApr framework resolves this dichotomy. By operating directly on the metric distance of the feature space rather than topological skeletons, ExApr retains the native distribution of the chemical space required to evaluate ID interpolation while systematically neutralizing the data leakage caused by exact and approximate feature collisions. When the ExApr filter is applied to the ID space (In-distribution ExApr), performance metrics drop to a mathematically honest, unbiased estimate of true in-domain generalization (e.g., AUC values normalizing to the 0.82–0.86 range).

Therefore, ExApr is not designed to replace scaffold splitting. Instead, ExApr serves as the necessary, bias-free counterpart for ID tasks. While scaffold splitting rigorously measures a model’s extrapolative potential for virtual screening, the ExApr framework measures its interpolative potential for lead optimization by systematically filtering out the memorization-based artifacts that artificially inflate performance. Together, these two evaluation paradigms provide a complete, bidirectional assessment of a model’s true predictive utility.

### 6.3 Performance Expectations and Training Dynamics

Previous studies have reported AUC values close to 1 in BBB prediction tasks, while others have shown AUC values of around 0.85 (see Table 1). This study suggests that models with an AUC of around 0.85 may have much stronger prediction capabilities than those with near-perfect AUC values if the latter were affected by overrepresentation bias. These findings highlight that model performance estimation is highly dependent on the presence of near-duplicate compounds in the test set. Therefore, a careful examination of the test set is essential to understand what can truly be expected from the developed models.

This work emphasizes the need for a renewed focus on how machine-learning models are evaluated for BBB permeability prediction. Furthermore, it raises the question of whether many other drug property prediction tasks could be severely affected by the same overrepresentation issue. Hence, we recommend the implementation of more restrictive data filtering criteria. As the community continues to adopt increasingly advanced models and integrate diverse chemical data sets, ensuring robust data curation and preprocessing is crucial. By addressing these challenges, we can mitigate the detrimental effects of overrepresentation bias and ultimately build more reliable, generalizable, and trustworthy predictive models.

Another relevant observation found in our work is that despite the performance differences observed in the test sets, the differences between training sets are minimal. Although the random training set contains approximately twice the number of samples compared to the exact and ExApr training sets (*N* = 5883 vs *N* = 3607), performance remains similar and, in some cases, even superior when the models are trained on the exact or ExApr data sets. This indicates that the reduced training size did not significantly impair the model’s learning capacity. Conversely, models trained on the largest data set (random) experienced a sharp performance drop when moved from the random test set to the ExApr test set. This asymmetry confirms that the observed performance drop is driven by the rigorous removal of near-duplicates from the evaluation set (bias mitigation), rather than a lack of training data.

We considered whether assigning lower sample weights to near-duplicate instances during training could serve as an alternative to data removal. However, our results indicate that overrepresentation bias is primarily an evaluation artifact, rather than a training dynamic issue. As observed in Tables 3 and 5, differences in the training set composition resulted in minimal performance variations. The magnitude of performance inflation was driven almost exclusively by the composition of the test set. Consequently, while downweighting duplicates during training might theoretically reduce gradient bias, it fails to correct the optimistic performance estimates generated when models are evaluated on test sets containing near-duplicates. To obtain a transparent and honest assessment of model predictive capabilities, we argue that removing these feature collisions from the evaluation set is the most rigorous approach.

A potential concern for practitioners is the computational expense of the proposed automatic detection method when applied to large-scale repositories. It is pertinent to decouple the cost of feature extraction from threshold optimization. The primary computational burden lies in calculating the nearest-neighbor distance for each compound. While naive pairwise comparison scales quadratically (*O*(*n* ^2^)), this step can be efficiently handled for massive data sets using modern approximate nearest neighbor indexing techniques, which are standard in high-dimensional chemoinformatics tasks. Crucially, the core innovation of this work—the Expectation-Maximization (EM) algorithm for threshold detection—incurs negligible computational overhead. The EM algorithm operates exclusively on the vector of nearest-neighbor distances (a 1D array of size *n*), rather than the full distance matrix. Thus, the proposed framework is highly scalable and introduces minimal latency beyond standard similarity search routines.

### 6.4 Feature Space Similarity vs True Chemical Representation

While we utilized ECFP as the primary feature space for bias detection due to its widespread adoption in QSAR and BBBP modeling, we must explicitly acknowledge the inherent mathematical limitations of this representation. However, among the specific feature representations evaluated in our modeling pipelines—which included 166 bit MACCS keys, 1D SMILES tokens, and global molecular descriptors—ECFP was unequivocally the most rigorous metric available for quantifying structural overlap. Unlike MACCS keys, which suffer from extreme hash degeneracy due to their short predefined bit-length, or global numerical descriptors, which can map completely different chemical scaffolds to similar numerical values (obscuring local topology), ECFP explicitly encodes local topological connectivity through circular atom environments. This makes it the most appropriate coordinate system for detecting the redundancy that our baseline models actually process.

To understand the scope of our methodology, it is crucial to distinguish between the *true chemical similarity* and *feature space similarity*. Our ExApr framework is intentionally designed to detect exact and approximate feature collisions within the specific representation space the machine learning models see—the numerical manifold from which they construct their decision boundaries. If we had utilized a “true” or more precise chemical representation to filter the data, we would have successfully distinguished complex structural differences, but doing so would have entirely masked the overlapping that occurs in the feature spaces utilized by the majority of current state-of-the-art models. This feature-space overlap is precisely the mechanism driving the overly optimistic performance estimates frequently reported in the literature. Therefore, to accurately audit the bias of models relying on 2D representations, it is an experimental necessity to measure redundancy within that same imperfect coordinate system.

Furthermore, we opted for 2D representations not only for direct comparability with previous studies but also because the source repositories provided structural data exclusively as 1D SMILES strings, lacking experimental 3D coordinates. Generating synthetic 3D conformers would introduce stochastic noise and uncertainty regarding the true bioactive conformations.

From the perspective of developing novel, highly robust predictive models, however, our reliance on ECFP introduces formal limitations that must be addressed in future works:

- Hash degeneracy: ECFPs rely on folding algorithms that map an essentially unbounded chemical space into a fixed-length bit array. This compression inherently introduces hash degeneracy; consequently, topologically distinct molecules can occasionally yield identical fingerprints because of bit collisions.
- Spatial and topological blind spots: Because ECFP compresses explicit graph connectivity and spatial geometry, it renders the current ExApr framework strictly a 1D/2D feature-leakage mitigation strategy. Two molecules that exhibit high similarity in a 2D ECFP space may possess distinct exact adjacency matrices or 3D conformations. Consequently, for models that rely on explicit spatial topologies (such as 3D-CNNs or equivariant graph networks), applying our 2D ECFP-based filtering may inadvertently destroy valid and required mathematical geometric variance, which is essential for model training.

To mitigate these limitations, future bias-aware frameworks must be adapted to the resolution of the target model. To reduce the overestimation of similarity caused by degenerate hash collisions in 2D pipelines, researchers should consider using concatenated arrays of multiple ECFP types to significantly enhance structural distinctiveness. Conversely, practitioners seeking to mitigate overrepresentation bias in 3D-aware architectures must substitute the 2D Jaccard distance with an appropriate 3D structural similarity metric to ensure that geometric data integrity is preserved.

### 6.5 Broader Implications

It is important to acknowledge the distinction between the class distribution of public benchmarks and real-world screening campaigns. Real-world high-throughput screening typically encounters much lower hit rates than those found in public benchmarks. We retained the naturally enriched distribution of the public repositories to maximize the structural diversity of the active class available for training. To mitigate the effects of this imbalance on evaluation, we relied on AUC-ROC (invariant to class priors) and macro F1-score (which weights the minority class equally). Crucially, the overrepresentation bias identified in this work operates independently of this prevalence. The performance inflation is driven by the nearest-neighbor leakage in the feature space, a mechanism that persists regardless of the global class ratio. Practitioners deploying these models in low-prevalence screening scenarios should anticipate that while the relative benefit of bias mitigation remains, the absolute precision metrics will naturally be adjusted to the lower base rates.

While this study empirically quantifies overrepresentation bias in BBBP prediction, the implications extend to the entire spectrum of QSAR and molecular property prediction. The root cause of this bias is intrinsic to medicinal chemistry: the synthesis of congeneric series during lead optimization. These dense clusters of analogs populate public repositories regardless of the end point being measured. Consequently, our ExApr framework—which filters redundancy based on the metric distance in the feature space rather than label distribution—provides a generalized methodology for establishing rigorous, bias-aware baselines across diverse chemoinformatics domains.

Finally, an important point to address is that while overrepresentation bias leads to exact and/or approximate instance collisions, the reverse is not always true. Sometimes feature collisions are present, but this does not necessarily mean the model is overfitting to specific areas of the feature space. The compound representation may position molecules in a feature space that is beneficial for the prediction task without compromising model prediction capabilities, even if exact or approximate collisions occur. However, considering common chemoinformatics representations—such as molecular descriptors, fingerprints, graph-based representations, or SMILES—this scenario is rare. It may be associated with specific feature extraction algorithms or representations obtained from the internal layers of models that have already undergone substantial data processing. That said, if these exact and approximate collisions in test sets are not caused by overrepresentation bias but rather result from a well-executed supervised feature extraction process, the performance differences across test sets should be minimal. In this case, the model would not be memorizing specific instances but rather extracting generalizable patterns from the compound representation, leading to consistent performance across different test sets.

## 7 Conclusions

In this work, we have developed multiple learning pipelines to investigate, within the BBB permeability prediction problem, the impact of a phenomenon that we identify as overrepresentation bias. This bias, caused by the presence of highly similar compounds in the data set, can lead to significant overestimation of model performance, giving the false impression that its prediction capabilities are far greater than they actually are.

We encourage the machine learning community—particularly the chemoinformatics community—not to overlook this issue. It is crucial to include additional test evaluations where exact and approximate instance collisions are properly addressed to provide meaningful context and improve the interpretability of the reported performance metrics.

## Supplementary Material



## Data Availability

The source data, along with the processed data presented in this study, can be found at our GitHub repository (https://github.com/bdslab-upv/bbbp-overrepresentation-bias). The code for both the overrepresentation-aware data splitting and the automatic approximate collision threshold calculation is available at our GitHub repository.

## Notes

Accuracy metrics with 95% nonparametric confidence intervals and percentage performance inflation for each developed pipeline; recall metrics with 95% nonparametric confidence intervals and percentage performance inflation; and specificity metrics with 95% nonparametric confidence intervals and percentage performance inflation (PDF)
