# Supplementary material for: Overrepresentation Bias Leads to Performance Overestimation in Blood–Brain Barrier Permeability Prediction Models: Characterization and Mitigation
Source: J Chem Inf Model. 2026 Jun 2;66(12):6837–54. doi: 10.1021/acs.jcim.5c02891 (PMC13292207; doi:10.1021/acs.jcim.5c02891)
Supplement: Supplementary file 1 [file ci5c02891_si_001.pdf]

Appendix A Additional performance estimation metrics

Next, we present complementary performance metrics calculated in this work. These metrics do not provide meaningful additional insights, and their values align with the findings reported in the Results and Discussion section. However, we include them for transparency and as additional evidence supporting our findings.

Table A1 Accuracy with 95% non-parametric confidence intervals shown in brackets, and the percentage performance inflation (Δ%) for each developed pipeline. Abbreviations: MolDesc., molecular descriptors; MACCS, molecular access system fingerprints; ECFP, extended-circular fingerprints; Graph, graph features; SMLILEStk, tokens from the simplified molecular input line entry system; RandFor, random forest; GradBst, gradient boosting; FeedFwd, feed forward deep neural network; SparEnc, sparse encoder; GraConv, graph convolutional network; ChBERTa, chemical bidirectional encoder representations from transformers; ExApr, exact and approximate.

Inputs	Model	Train set	Test set			Δ%
			Random	Exact	ExApr	
MolDesc	RandFor	Random	0.88 [0.86, 0.90]	0.77 [0.74, 0.80]	0.77 [0.74, 0.80]	14.20 [7.50, 21.40]
MolDesc	RandFor	Exact	0.88 [0.86, 0.90]	0.77 [0.74, 0.80]	0.77 [0.74, 0.80]	14.30 [7.50, 21.60]
MolDesc	RandFor	ExaApr	0.88 [0.86, 0.90]	0.77 [0.74, 0.80]	0.77 [0.74, 0.80]	14.00 [7.20, 20.80]
MolDesc	GradBst	Random	0.90 [0.88, 0.92]	0.79 [0.77, 0.82]	0.79 [0.76, 0.82]	13.90 [7.80, 20.80]
MolDesc	GradBst	Exact	0.90 [0.88, 0.92]	0.79 [0.76, 0.82]	0.79 [0.76, 0.81]	14.50 [8.20, 21.30]
MolDesc	GradBst	ExaApr	0.90 [0.88, 0.92]	0.78 [0.75, 0.81]	0.78 [0.75, 0.80]	15.40 [9.10, 22.30]
MolDesc	FeedFwd	Random	0.83 [0.80, 0.85]	0.72 [0.69, 0.76]	0.72 [0.69, 0.75]	14.70 [6.60, 23.10]
MolDesc	FeedFwd	Exact	0.81 [0.79, 0.84]	0.72 [0.69, 0.75]	0.72 [0.69, 0.75]	13.20 [4.70, 22.30]
MolDesc	FeedFwd	ExaApr	0.81 [0.78, 0.83]	0.72 [0.69, 0.75]	0.72 [0.69, 0.75]	12.10 [4.00, 21.00]
MACCS	RandFor	Random	0.89 [0.87, 0.91]	0.77 [0.75, 0.80]	0.77 [0.74, 0.80]	18.30 [10.70, 26.80]
MACCS	RandFor	Exact	0.89 [0.87, 0.91]	0.78 [0.75, 0.81]	0.78 [0.75, 0.81]	15.40 [7.30, 23.80]
MACCS	RandFor	ExaApr	0.88 [0.86, 0.90]	0.77 [0.75, 0.80]	0.77 [0.75, 0.80]	16.50 [8.60, 24.90]
MACCS	GradBst	Random	0.89 [0.86, 0.91]	0.78 [0.75, 0.80]	0.78 [0.75, 0.81]	17.90 [10.90, 25.80]
MACCS	GradBst	Exact	0.90 [0.88, 0.92]	0.77 [0.75, 0.80]	0.77 [0.74, 0.80]	16.90 [9.80, 24.30]
MACCS	GradBst	ExaApr	0.89 [0.87, 0.91]	0.77 [0.74, 0.80]	0.77 [0.74, 0.80]	17.00 [10.20, 24.40]
MACCS	SparEnc	Random	0.88 [0.86, 0.90]	0.76 [0.73, 0.79]	0.76 [0.73, 0.79]	19.20 [11.80, 27.70]
MACCS	SparEnc	Exact	0.88 [0.86, 0.90]	0.76 [0.73, 0.79]	0.76 [0.73, 0.79]	18.50 [10.60, 26.70]
MACCS	SparEnc	ExaApr	0.88 [0.86, 0.90]	0.74 [0.71, 0.77]	0.74 [0.71, 0.77]	21.00 [13.20, 29.20]
ECFP	RandFor	Random	0.86 [0.84, 0.88]	0.73 [0.69, 0.76]	0.73 [0.69, 0.76]	15.10 [8.40, 22.30]
ECFP	RandFor	Exact	0.83 [0.81, 0.86]	0.72 [0.69, 0.76]	0.72 [0.69, 0.75]	14.70 [8.10, 21.50]
ECFP	RandFor	ExaApr	0.83 [0.81, 0.86]	0.72 [0.68, 0.75]	0.72 [0.69, 0.75]	14.10 [7.50, 20.90]
ECFP	GradBst	Random	0.88 [0.86, 0.90]	0.75 [0.72, 0.78]	0.75 [0.72, 0.78]	14.30 [7.30, 21.10]
ECFP	GradBst	Exact	0.88 [0.86, 0.90]	0.75 [0.72, 0.78]	0.75 [0.72, 0.78]	16.10 [9.40, 22.80]
ECFP	GradBst	ExaApr	0.88 [0.86, 0.90]	0.75 [0.72, 0.78]	0.75 [0.73, 0.78]	15.50 [8.70, 22.60]
ECFP	SparEnc	Random	0.87 [0.85, 0.89]	0.73 [0.70, 0.76]	0.73 [0.70, 0.76]	16.20 [9.50, 23.70]
ECFP	SparEnc	Exact	0.87 [0.85, 0.89]	0.74 [0.71, 0.77]	0.73 [0.70, 0.77]	16.40 [9.00, 23.50]
ECFP	SparEnc	ExaApr	0.87 [0.85, 0.89]	0.72 [0.69, 0.75]	0.72 [0.69, 0.75]	19.50 [12.10, 27.20]
Graph	GraConv	Random	0.88 [0.86, 0.90]	0.75 [0.72, 0.78]	0.75 [0.73, 0.78]	17.00 [9.90, 24.50]
Graph	GraConv	Exact	0.87 [0.85, 0.89]	0.73 [0.70, 0.76]	0.73 [0.70, 0.76]	19.20 [11.20, 27.30]
Graph	GraConv	ExaApr	0.87 [0.85, 0.89]	0.73 [0.70, 0.76]	0.73 [0.70, 0.76]	18.60 [11.20, 27.10]
SMILEStk	ChBERTa	Random	0.88 [0.85, 0.90]	0.77 [0.73, 0.79]	0.77 [0.74, 0.79]	14.40 [7.40, 21.70]
SMILEStk	ChBERTa	Exact	0.84 [0.81, 0.86]	0.76 [0.73, 0.79]	0.76 [0.73, 0.79]	10.60 [3.30, 18.30]
SMILEStk	ChBERTa	ExaApr	0.86 [0.83, 0.88]	0.77 [0.74, 0.80]	0.77 [0.74, 0.80]	11.10 [4.00, 18.80]

Table A2 Recall with 95% non-parametric confidence intervals shown in brackets, and the percentage performance inflation (Δ%) for each developed pipeline. Abbreviations: MolDesc., molecular descriptors; MACCS, molecular access system fingerprints; ECFP, extended-circular fingerprints; Graph, graph features; SMLILEStk, tokens from the simplified molecular input line entry system; RandFor, random forest; GradBst, gradient boosting; FeedFwd, feed forward deep neural network; SparEnc, sparse encoder; GraConv, graph convolutional network; ChBERTa, chemical bidirectional encoder representations from transformers; ExApr, exact and approximate.

Inputs	Model	Train set	Test set			Δ%
			Random	Exact	ExApr	
MolDesc	RandFor	Random	0.93 [0.90, 0.95]	0.89 [0.86, 0.92]	0.89 [0.86, 0.92]	4.30 [-1.50, 10.40]
MolDesc	RandFor	Exact	0.92 [0.89, 0.94]	0.88 [0.84, 0.91]	0.87 [0.84, 0.90]	5.00 [-1.20, 11.90]
MolDesc	RandFor	ExaApr	0.92 [0.89, 0.94]	0.88 [0.85, 0.91]	0.88 [0.85, 0.91]	4.60 [-1.80, 11.20]
MolDesc	GradBst	Random	0.92 [0.89, 0.94]	0.87 [0.84, 0.90]	0.87 [0.84, 0.90]	5.30 [-1.00, 12.00]
MolDesc	GradBst	Exact	0.93 [0.90, 0.95]	0.89 [0.86, 0.91]	0.89 [0.86, 0.92]	4.50 [-1.50, 10.70]
MolDesc	GradBst	ExaApr	0.93 [0.90, 0.95]	0.87 [0.84, 0.90]	0.87 [0.84, 0.90]	6.30 [0.10, 12.60]
MolDesc	FeedFwd	Random	0.85 [0.82, 0.89]	0.80 [0.76, 0.83]	0.79 [0.76, 0.83]	8.00 [-1.10, 17.10]
MolDesc	FeedFwd	Exact	0.80 [0.76, 0.83]	0.74 [0.70, 0.78]	0.74 [0.69, 0.78]	7.90 [-2.70, 19.90]
MolDesc	FeedFwd	ExaApr	0.81 [0.77, 0.84]	0.77 [0.73, 0.81]	0.77 [0.73, 0.80]	5.20 [-4.10, 15.70]
MACCS	RandFor	Random	0.92 [0.90, 0.94]	0.86 [0.82, 0.89]	0.86 [0.82, 0.89]	12.60 [3.60, 22.10]
MACCS	RandFor	Exact	0.92 [0.89, 0.94]	0.85 [0.81, 0.88]	0.85 [0.81, 0.88]	19.50 [6.30, 34.30]
MACCS	RandFor	ExaApr	0.91 [0.88, 0.93]	0.84 [0.81, 0.88]	0.84 [0.81, 0.88]	21.80 [8.20, 37.20]
MACCS	GradBst	Random	0.91 [0.88, 0.93]	0.82 [0.79, 0.86]	0.82 [0.78, 0.86]	11.60 [3.60, 19.90]
MACCS	GradBst	Exact	0.91 [0.88, 0.93]	0.82 [0.78, 0.85]	0.82 [0.78, 0.85]	12.30 [4.50, 21.30]
MACCS	GradBst	ExaApr	0.91 [0.88, 0.93]	0.81 [0.78, 0.85]	0.82 [0.78, 0.85]	11.90 [4.00, 20.40]
MACCS	SparEnc	Random	0.91 [0.88, 0.93]	0.81 [0.77, 0.85]	0.81 [0.78, 0.85]	14.40 [5.30, 23.60]
MACCS	SparEnc	Exact	0.90 [0.87, 0.93]	0.81 [0.77, 0.84]	0.81 [0.78, 0.85]	10.20 [2.10, 19.20]
MACCS	SparEnc	ExaApr	0.89 [0.86, 0.92]	0.76 [0.72, 0.80]	0.76 [0.72, 0.80]	11.30 [3.30, 19.70]
ECFP	RandFor	Random	0.87 [0.84, 0.90]	0.78 [0.74, 0.81]	0.78 [0.74, 0.81]	7.60 [0.90, 14.40]
ECFP	RandFor	Exact	0.78 [0.74, 0.81]	0.65 [0.61, 0.70]	0.65 [0.61, 0.69]	8.20 [1.10, 15.40]
ECFP	RandFor	ExaApr	0.77 [0.73, 0.81]	0.63 [0.59, 0.68]	0.63 [0.59, 0.68]	7.90 [0.80, 15.00]
ECFP	GradBst	Random	0.90 [0.87, 0.93]	0.81 [0.77, 0.84]	0.81 [0.77, 0.84]	10.60 [2.40, 18.90]
ECFP	GradBst	Exact	0.89 [0.87, 0.92]	0.80 [0.76, 0.83]	0.80 [0.76, 0.83]	11.10 [3.40, 19.20]
ECFP	GradBst	ExaApr	0.90 [0.88, 0.93]	0.81 [0.77, 0.84]	0.81 [0.77, 0.84]	11.10 [3.30, 19.30]
ECFP	SparEnc	Random	0.89 [0.86, 0.92]	0.78 [0.74, 0.82]	0.78 [0.74, 0.82]	11.60 [3.70, 19.70]
ECFP	SparEnc	Exact	0.89 [0.86, 0.92]	0.81 [0.78, 0.85]	0.81 [0.77, 0.85]	11.40 [2.70, 19.30]
ECFP	SparEnc	ExaApr	0.90 [0.88, 0.93]	0.81 [0.78, 0.85]	0.81 [0.78, 0.85]	17.30 [8.10, 26.60]
Graph	GraConv	Random	0.87 [0.84, 0.90]	0.75 [0.71, 0.79]	0.75 [0.71, 0.79]	15.50 [5.60, 26.50]
Graph	GraConv	Exact	0.87 [0.84, 0.90]	0.75 [0.71, 0.79]	0.75 [0.71, 0.80]	16.40 [5.90, 26.40]
Graph	GraConv	ExaApr	0.87 [0.84, 0.90]	0.76 [0.72, 0.80]	0.75 [0.71, 0.80]	15.10 [5.70, 25.60]
SMILEStk	ChBERTa	Random	0.89 [0.86, 0.92]	0.79 [0.76, 0.83]	0.79 [0.76, 0.83]	12.10 [4.00, 21.00]
SMILEStk	ChBERTa	Exact	0.85 [0.81, 0.88]	0.78 [0.74, 0.82]	0.78 [0.74, 0.81]	9.10 [0.10, 18.80]
SMILEStk	ChBERTa	ExaApr	0.87 [0.84, 0.90]	0.80 [0.77, 0.84]	0.80 [0.76, 0.84]	8.40 [-0.10, 17.70]

Table A3 Specificity with 95% non-parametric confidence intervals shown in brackets, and the percentage performance inflation (Δ%) for each developed pipeline. Abbreviations: MolDesc., molecular descriptors; MACCS, molecular access system fingerprints; ECFP, extended-circular fingerprints; Graph, graph features; SMLILEStk, tokens from the simplified molecular input line entry system; RandFor, random forest; GradBst, gradient boosting; FeedFwd, feed forward deep neural network; SparEnc, sparse encoder; GraConv, graph convolutional network; ChBERTa, chemical bidirectional encoder representations from transformers; ExApr, exact and approximate.

Inputs	Model	Train set	Test set			Δ%
			Random	Exact	ExApr	
MolDesc	RandFor	Random	0.83 [0.79, 0.87]	0.63 [0.58, 0.68]	0.63 [0.58, 0.68]	31.10 [16.30, 48.20]
MolDesc	RandFor	Exact	0.84 [0.80, 0.88]	0.65 [0.60, 0.69]	0.65 [0.60, 0.70]	29.10 [14.90, 46.10]
MolDesc	RandFor	ExaApr	0.83 [0.80, 0.87]	0.65 [0.60, 0.69]	0.65 [0.60, 0.69]	29.30 [14.80, 45.70]
MolDesc	GradBst	Random	0.88 [0.85, 0.92]	0.70 [0.65, 0.74]	0.70 [0.65, 0.75]	26.30 [14.10, 40.90]
MolDesc	GradBst	Exact	0.87 [0.84, 0.91]	0.67 [0.62, 0.72]	0.67 [0.62, 0.72]	30.10 [16.80, 45.70]
MolDesc	GradBst	ExaApr	0.86 [0.82, 0.90]	0.67 [0.62, 0.71]	0.67 [0.61, 0.71]	29.60 [15.60, 45.70]
MolDesc	FeedFwd	Random	0.80 [0.76, 0.84]	0.64 [0.59, 0.69]	0.64 [0.59, 0.69]	24.60 [10.00, 42.80]
MolDesc	FeedFwd	Exact	0.83 [0.80, 0.87]	0.70 [0.65, 0.74]	0.70 [0.65, 0.74]	19.80 [7.40, 34.40]
MolDesc	FeedFwd	ExaApr	0.81 [0.77, 0.85]	0.67 [0.62, 0.72]	0.67 [0.62, 0.72]	21.40 [7.50, 37.10]
MACCS	RandFor	Random	0.85 [0.81, 0.89]	0.67 [0.63, 0.72]	0.67 [0.63, 0.72]	26.20 [12.80, 41.50]
MACCS	RandFor	Exact	0.86 [0.83, 0.89]	0.69 [0.65, 0.74]	0.69 [0.65, 0.74]	11.20 [2.50, 20.40]
MACCS	RandFor	ExaApr	0.85 [0.82, 0.89]	0.69 [0.65, 0.74]	0.69 [0.65, 0.74]	11.50 [3.20, 20.40]
MACCS	GradBst	Random	0.86 [0.82, 0.89]	0.72 [0.67, 0.77]	0.72 [0.68, 0.77]	27.10 [13.80, 41.70]
MACCS	GradBst	Exact	0.88 [0.85, 0.91]	0.72 [0.68, 0.76]	0.72 [0.67, 0.76]	22.80 [10.70, 35.80]
MACCS	GradBst	ExaApr	0.88 [0.84, 0.91]	0.72 [0.68, 0.77]	0.72 [0.68, 0.77]	24.20 [11.40, 39.20]
MACCS	SparEnc	Random	0.86 [0.82, 0.89]	0.70 [0.65, 0.74]	0.70 [0.65, 0.74]	26.00 [13.40, 41.60]
MACCS	SparEnc	Exact	0.86 [0.82, 0.89]	0.70 [0.66, 0.75]	0.69 [0.65, 0.74]	30.90 [16.30, 47.20]
MACCS	SparEnc	ExaApr	0.87 [0.83, 0.90]	0.71 [0.67, 0.75]	0.71 [0.66, 0.75]	36.60 [20.80, 54.40]
ECFP	RandFor	Random	0.84 [0.81, 0.88]	0.67 [0.62, 0.71]	0.67 [0.62, 0.72]	26.40 [13.20, 41.50]
ECFP	RandFor	Exact	0.90 [0.87, 0.93]	0.81 [0.77, 0.85]	0.81 [0.77, 0.85]	24.20 [11.30, 37.80]
ECFP	RandFor	ExaApr	0.91 [0.88, 0.94]	0.82 [0.78, 0.85]	0.82 [0.78, 0.85]	23.20 [10.40, 37.00]
ECFP	GradBst	Random	0.86 [0.82, 0.89]	0.68 [0.63, 0.72]	0.68 [0.63, 0.72]	19.10 [7.70, 32.30]
ECFP	GradBst	Exact	0.86 [0.83, 0.90]	0.70 [0.66, 0.75]	0.70 [0.66, 0.75]	22.60 [11.20, 35.70]
ECFP	GradBst	ExaApr	0.86 [0.82, 0.89]	0.69 [0.65, 0.74]	0.69 [0.64, 0.74]	21.40 [9.90, 33.30]
ECFP	SparEnc	Random	0.85 [0.81, 0.89]	0.68 [0.63, 0.72]	0.67 [0.62, 0.72]	22.80 [10.60, 36.70]
ECFP	SparEnc	Exact	0.84 [0.81, 0.88]	0.65 [0.60, 0.69]	0.64 [0.60, 0.69]	23.30 [11.10, 37.90]
ECFP	SparEnc	ExaApr	0.83 [0.80, 0.87]	0.61 [0.56, 0.66]	0.61 [0.56, 0.66]	22.60 [10.90, 36.00]
Graph	GraConv	Random	0.90 [0.87, 0.93]	0.76 [0.72, 0.80]	0.76 [0.71, 0.80]	19.00 [8.60, 30.40]
Graph	GraConv	Exact	0.86 [0.83, 0.90]	0.70 [0.66, 0.75]	0.70 [0.66, 0.75]	22.60 [11.10, 35.60]
Graph	GraConv	ExaApr	0.87 [0.84, 0.90]	0.70 [0.66, 0.75]	0.70 [0.66, 0.75]	23.00 [11.60, 36.30]
SMILEStk	ChBERTa	Random	0.86 [0.82, 0.89]	0.73 [0.69, 0.78]	0.73 [0.69, 0.78]	17.40 [6.30, 29.00]
SMILEStk	ChBERTa	Exact	0.82 [0.78, 0.86]	0.73 [0.69, 0.78]	0.73 [0.69, 0.78]	12.40 [0.90, 24.90]
SMILEStk	ChBERTa	ExaApr	0.85 [0.81, 0.88]	0.74 [0.69, 0.78]	0.74 [0.70, 0.78]	14.70 [3.20, 27.00]
